# Sodium Butyrate Attenuates Sevoflurane‐Induced Impaired Myelination and Neurobehavioral Deficits in Neonatal Mice via the H3K9ac/BDNF/TrkB Pathway

**DOI:** 10.1002/cns.71026

**Published:** 2026-07-09

**Authors:** Chang Liu, Xinyao Bai, Xinyue Liu, Jinjie Li, Ruizhu Liu, Jinlan Jiang, Guoqing Zhao

**Affiliations:** ^1^ Department of Anaesthesia China‐Japan Union Hospital of Jilin University Changchun China; ^2^ Department of Anaesthesia The First Hospital of Jilin University Changchun China; ^3^ Department of Scientific Research Center China‐Japan Union Hospital of Jilin University Changchun China; ^4^ Jilin University Changchun China

**Keywords:** myelination, neonatal mice, neurobehavioral deficits, sevoflurane, sodium butyrate

## Abstract

**Background:**

Early‐life exposure to sevoflurane is considered an important risk factor for abnormal neurodevelopment and may lead to impaired myelination and neurobehavioral deficits in neonatal mice. Sodium butyrate (NaB), a short‐chain fatty acid (SCFA) with epigenetic regulatory activity, has shown neuroprotective effects in various neurological disorders. However, the role of NaB in sevoflurane‐induced myelination impairment and the underlying molecular mechanisms remain unclear.

**Methods:**

An in vivo neonatal mouse model of sevoflurane exposure was established to investigate its effects on myelination and neurobehavioral function. NaB intervention was then administered to evaluate its potential to ameliorate sevoflurane‐induced myelination impairment and neurobehavioral deficits. In vitro, human oligodendrocyte precursor cells (HOPCs) were directly treated with different concentrations of NaB to examine its effects on cell proliferation, migration, differentiation, and related molecular signaling pathways.

**Results:**

Repeated exposure to sevoflurane during the critical period of brain development in neonatal mice inhibited the proliferation and differentiation of oligodendrocyte precursor cells (OPCs), impaired myelination, and led to neurobehavioral deficits. NaB intervention ameliorated sevoflurane‐induced myelination impairment and neurobehavioral dysfunction by regulating histone H3 lysine 9 acetylation (H3K9ac), upregulating brain‐derived neurotrophic factor (BDNF) expression, promoting tropomyosin receptor kinase B (TrkB) phosphorylation, and increasing phosphorylated TrkB (p‐TrkB) levels.

**Conclusions:**

Repeated sevoflurane exposure in neonatal mice inhibited OPC proliferation and differentiation, impaired myelination, and caused neurobehavioral deficits. NaB ameliorated these impairments by regulating H3K9ac modification and increasing BDNF and p‐TrkB levels.

## Graphical abstract

Repeated exposure to sevoflurane in neonatal mice leads to impaired myelin development and neurobehavioral dysfunction, a process closely associated with reduced butyrate levels in vivo. Sodium butyrate supplementation effectively ameliorates these sevoflurane‐induced abnormalities by downregulating HDAC3 and HDAC8 expression, enhancing H3K9ac levels, and activating the BDNF/TrkB signaling pathway.

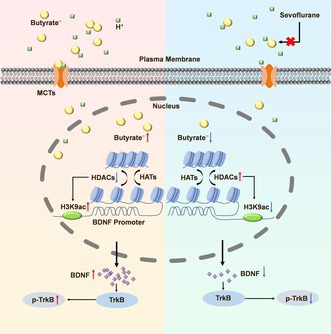

**Abbreviations**
AD: Alzheimer's disease
ANOVA: analysis of variance
APC: adenomatous polyposis coli
BCA: bicinchoninic acid
BDNF: brain‐derived neurotrophic factor
BrdU: 5‐bromo‐2‐deoxyuridine
BSA: bovine serum albumin
CCK‐8: cell counting kit‐8
cDNA: complementary deoxyribonucleic acid
ChIP: chromatin immunoprecipitation
CNPase: 2′,3′‐cyclic nucleotide 3′‐phosphodiesterase
CNS: central nervous system
DAPI: 4′,6‐diamidino‐2‐phenylindole
DNA: deoxyribonucleic acid
ECL: enhanced chemiluminescence
EDTA: ethylenediaminetetraacetic acid
EdU: (5‐ethynyl‐2′‐deoxyuridine)
ELISA: enzyme‐linked immunosorbent assay
FBS: fetal bovine serum
GA: general anesthesia
GC–MS: gas chromatography–mass spectrometry
H3K9ac: histone H3 lysine 9 acetylation
HATs: histone acetyltransferases
HDACs: histone deacetylases
HOPCs: human oligodendrocyte precursor cells
HRP: horseradish peroxidase
ICC‐F: immunocytochemistry with fluorescence detection
IF: immunofluorescence
KO: knockout
LFB: Luxol fast blue
LiCl: lithium chloride
MBP: myelin basic protein
MWM: Morris water maze
Myrf: myelin regulatory factor
NaB: sodium butyrate
NORT: novel object recognition test
Olig2: oligodendrocyte transcription factor 2
OLs: oligodendrocytes
OLT: object location test
OPCDM: oligodendrocyte precursor cell differentiation medium
OPCDS: oligodendrocyte precursor cell differentiation supplement
OPCGS: oligodendrocyte precursor cell growth supplement
OPCM: oligodendrocyte precursor cell medium
OPCs: oligodendrocyte precursor cells
P/S: penicillin/streptomycin solution
p75NTR: p75 neurotrophin receptor
PBS: phosphate‐buffered saline
PDGFRα: platelet‐derived growth factor receptor alpha
PFA: paraformaldehyde
PLL: poly‐L‐lysine
PNS: peripheral nervous system
p‐TrkB: phosphorylated TrkB
PVDF: polyvinylidene fluoride
QC: quality control
RT‐qPCR: reverse transcription quantitative polymerase chain reaction
SCFAs: short‐chain fatty acids
SDS‐PAGE: sodium dodecyl sulfate‐polyacrylamide gel electrophoresis
SOX10: SRY‐box transcription factor 10
TBST: Tris‐buffered saline containing Tween‐20
TE: Tris‐EDTA
TEM: transmission electron microscopy
TfR1: transferrin receptor 1
TrkB: tropomyosin receptor kinase B
WB: western blot
ZDHHC5: zinc finger DHHC‐type palmitoyltransferase 5

## 1 Introduction

General anesthesia (GA) is an indispensable life‐support modality in modern surgery, and millions of infants and young children worldwide receive GA each year for diagnostic and therapeutic purposes [1]. Sevoflurane, owing to its favorable safety profile, rapid onset, and good tolerability, has become a widely used inhalational general anesthetic in clinical practice [2]. Notably, accumulating evidence has indicated that sevoflurane has potential neurotoxicity. Its adverse effects are particularly pronounced in vulnerable populations, especially during central nervous system (CNS) development or under neurodegenerative conditions, and are clinically manifested mainly as neurobehavioral deficits [3, 4, 5]. The safety of anesthesia in infants and young children is a major health and social concern. In rodents, myelination begins around postnatal Day 3 (P3) and continues until approximately 8 weeks of age, with P7–P21 representing a critical window for OPC differentiation [6], This stage roughly corresponds to 1–6 months of age in human infants. Exposure to inhalational general anesthetics during this period may inhibit OPC differentiation and disrupt myelin protein synthesis, thereby leading to delayed myelination and structural abnormalities and potentially causing long‐term impairment of neural conduction [7].

In our previous study, we found that neonatal mice exposed to 3% sevoflurane for 2 h daily over three consecutive days developed gut microbiota dysbiosis, characterized by significant reductions in the butyrate‐producing genera Dwaynesavagella and Eubacterium_F, along with decreased intestinal levels of multiple SCFAs, among which butyrate showed the greatest and most sustained decline [8]. Previous studies have shown that antibiotic‐induced gut microbiota dysbiosis and reduced SCFA levels in mice can inhibit the differentiation of immature oligodendrocytes (OLs), whereas butyrate supplementation can partially reverse these changes [9], However, the underlying mechanisms remain unclear.

Histones are major protein components of eukaryotic chromatin and are essential for the regulation of gene expression and neurodevelopment. They interact closely with deoxyribonucleic acid (DNA) to form nucleosomes and regulate chromatin accessibility through epigenetic modifications, thereby influencing the transcriptional activity of neurodevelopment‐related genes. Histone acetylation, as one of the key epigenetic modifications, is dynamically regulated mainly by histone acetyltransferases (HATs) and histone deacetylases (HDACs) and is closely associated with neuronal differentiation, synaptic plasticity, and learning and memory [10]. As a natural HDAC inhibitor, butyrate can increase histone acetylation levels and promote chromatin relaxation, thereby providing binding sites for transcription factors and chromatin‐remodeling complexes, which facilitates the activation of specific gene expression and the fine regulation of neural network function [11]. BDNF is a neurotrophin closely associated with neuronal survival and synaptic plasticity. By binding to its high‐affinity receptor TrkB and promoting its phosphorylation, BDNF activates downstream signaling pathways [12], thereby influencing multiple key processes in neurodevelopment [13].

Although previous studies have shown that butyrate exerts neuroprotective effects by regulating histone acetylation, direct evidence is still lacking as to whether it acts as a key mediator linking sevoflurane exposure to myelination impairment. Based on this, we hypothesized that sevoflurane exposure may suppress OPC proliferation and differentiation by reducing endogenous butyrate levels, thereby leading to abnormal myelination; conversely, NaB supplementation may promote OPC proliferation and differentiation by enhancing histone acetylation, upregulating BDNF expression, and increasing p‐TrkB levels, thereby ameliorating myelination impairment and alleviating neurobehavioral deficits. From the perspective of the “gut metabolite‐epigenetic regulation‐myelination” axis, this study establishes a novel mechanistic link and provides a new perspective for understanding anesthetic‐related neurodevelopmental injury.

## 2 Materials and Methods

### 2.1 Animals and Experimental Design

Pregnant C57BL/6J mice at gestational Day 18 (G18) were purchased from Jilin Qianhe Model Biotechnology Co. Ltd. (license No. SYXK [Ji] 2021‐0003). The animals were housed in a strictly controlled barrier environment with an ambient temperature of 22°C–24°C, relative humidity of 40%–50%, and a 12 h/12 h light–dark cycle, with free access to food and water. After natural delivery, neonatal pups from the same litter were randomly assigned to different groups, with balanced sex ratios across groups. A total of 60 neonatal mice were included in this study, and the experiments were initiated on P6.

This study consisted of two parts. Experiment 1 was designed to evaluate the effects of sevoflurane exposure on neonatal mice and included the Con group and the Sev group, with 10 mice in each group. Experiment 2 was conducted to assess the intervention effects of NaB and ANA‐12 and included the Con+NS + Veh group, Sev + NS + Veh group, Sev + NaB+Veh group, and Sev + NaB + ANA‐12 group, with 10 mice in each group. The specific treatment protocols for each group are shown in Table 1.

**TABLE 1:** Experimental groups and treatment protocol.

Group	Gas exposure (P6–P8, 2 h/day)	Oral gavage (P9–P29, once daily)	i.p. injection (P9–P29, once daily)
Con	Carrier gas	None	None
Sev	3% sevoflurane	None	None
Con + NS + Veh	Carrier gas	Normal saline (NS)	Vehicle (Veh)
Sev + NS + Veh	3% sevoflurane	Normal saline (NS)	Vehicle (Veh)
Sev + NaB + Veh	3% sevoflurane	NaB (300 mg/kg)	Vehicle (Veh)
Sev + NaB + ANA‐12	3% sevoflurane	NaB (300 mg/kg)	ANA‐12 (0.5 mg/kg)

*Note:* The carrier gas consisted of 60% N_2_ and 40% O_2_. Vehicle consisted of DMSO, PEG300, 5% Tween 80, and 45% saline. NaB (Cat# HY‐B0350A, MedChemExpress, USA) and ANA‐12 (Cat# HY‐12497, MedChemExpress, USA).

Sevoflurane exposure was performed using a small‐animal anesthesia machine (R630, RWD Life Science Co. Ltd., China) to conceptually simulate repeated anesthetic exposure in infants and young children [14]. Briefly, mice were first induced in a Plexiglas chamber with oxygen delivered at 5 L/min for 3–5 min. After the loss of the righting reflex, the oxygen flow rate was reduced to 2 L/min, and anesthesia was maintained for 2 h. During anesthesia, sevoflurane, oxygen, and carbon dioxide concentrations were continuously monitored using a multigas monitor (Ohmeda S/5 Compact, Datex‐Ohmeda, USA), while heart rate and body temperature were monitored using a veterinary monitor (GT6800VET, Jeruitai, China). Body temperature was maintained at 36.5°C ± 0.5°C with a heating pad. After anesthetic exposure, the mice were returned to their home cages with the dam once the righting reflex had recovered. All animals survived.

In addition, three mice were randomly selected from each group and intraperitoneally injected with 5‐bromo‐2‐deoxyuridine (BrdU; 50 mg/kg; Cat# HY‐15910; MedChemExpress, USA) once daily for five consecutive days from P37 to P41. All mice underwent behavioral testing between P30 and P42 and were euthanized after completion of the experiments.

### 2.2 Behavior Tests

All behavioral tests were performed between 9:00 and 18:00. After each test, the apparatus was cleaned with 75% ethanol to eliminate olfactory interference. Mouse behavior was analyzed and relevant data were recorded using animal behavior video analysis software (SANS Biological Technology Co. Ltd., China).

#### 2.2.1 Rotarod Test

The rotarod test was performed on P30–P33 to evaluate motor coordination, balance, and motor learning in mice. Mice underwent pretraining for 3 days before the formal test, with two sessions per day and 2 min per session, at a fixed speed of 4 r/min. The interval between the two training sessions was 15 min. During the formal test, the accelerating mode was used, with the rotation speed gradually increasing from 4 r/min to 40 r/min over 3 min and then maintained until the end of the 5 min test period. The latency from the onset of rotation to the first fall was recorded. Each mouse was tested three times, and the mean value of the three trials was used for final analysis. The trial was terminated if the mouse fell off the rod or passively rotated on the rod for two consecutive revolutions.

#### 2.2.2 Beam Walking Test

The beam walking test was conducted on P30–P33 to assess fine motor control, balance, and limb coordination in mice. The apparatus consisted of a horizontally placed beam measuring 80 cm in length and 0.5 cm in width, elevated 50 cm above the floor, with a black escape box attached to one end and a soft pad placed underneath. Mice underwent pretraining for 3 days before the formal test, with two sessions per day and a 15 min interval between sessions. During training, home‐cage bedding was placed in the escape box to encourage forward movement. Each mouse was placed at the starting end of the beam and allowed to traverse to the goal box voluntarily, with gentle guidance provided when necessary. During the formal test, the time required for the mouse to traverse from the starting point to the endpoint and the number of foot slips during the task were recorded. Each mouse was tested three times, and the mean value of the three trials was used for statistical analysis.

#### 2.2.3 Object Location Test (OLT) and Novel Object Recognition Test (NORT)

The OLT and NORT were performed on P34–P37 to assess short‐term and long‐term memory in mice. Mice were habituated to the testing room for 30 min before the experiment. During the habituation phase, mice were allowed to freely explore the open‐field arena for 6 min, repeated three times with 20 min intervals. During the testing phase, objects A and B were first placed 7 cm from the walls of the arena, and the mice were allowed to explore for 10 min. After a 30 min interval, object B was moved to a new location (B*), and the mice were reintroduced for exploration. Object A was then replaced with object C, followed by another exploration session. After a 1‐day interval, objects D and E were placed in the arena for 10 min of exploration. On the following day, object E was replaced with object F, and the mice were allowed to explore again.

#### 2.2.4 Morris Water Maze (MWM) Test

The MWM test was performed on P38–P42 to assess learning and memory in mice. The escape platform was positioned 1 cm below the water surface. The place navigation test was conducted over four consecutive days. If a mouse found the platform within 60 s, it was allowed to remain on the platform for 10 s. If the mouse failed to find the platform within 60 s, it was gently guided to the platform by the experimenter and allowed to stay there for 30 s. On Day 5, the platform was removed, and a 60 s probe trial was performed. After each trial, the mice were placed in a warming cage for 5 min to dry before being returned to their home cages.

### 2.3 Histological and Morphological Analyses

#### 2.3.1 Luxol Fast Blue (LFB) Staining

After fixation in 4% paraformaldehyde (PFA) for 24 h, mouse brain tissues were dehydrated through a graded ethanol series, cleared in xylene, and infiltrated with paraffin, followed by embedding and sectioning into 5 μm‐thick coronal brain sections. After deparaffinization and rehydration, the sections were incubated overnight at 60°C with LFB staining solution (Sigma‐Aldrich, USA). Following staining, the sections were differentiated in 70% ethanol, rehydrated, dehydrated, cleared, and coverslipped. Images were then observed and captured under a light microscope (Nikon Eclipse Ni‐U, Japan).

#### 2.3.2 Transmission Electron Microscopy (TEM)

Mouse brain tissues were initially prefixed in a mixed fixative containing 3% PFA and 1% glutaraldehyde, and then transferred to 2% glutaraldehyde for overnight fixation at 4°C. After rinsing with phosphate‐buffered saline (PBS), the tissues were postfixed in 1% osmium tetroxide for 1.5 h at 4°C. The samples were subsequently dehydrated through a graded ethanol series, infiltrated with propylene oxide, and embedded in epoxy resin (Epon 812), followed by polymerization at 60°C for 24 h. Ultrathin sections were prepared using an ultramicrotome (Leica UC7), double‐stained with uranyl acetate and lead citrate, and observed and imaged under a transmission electron microscope (Hitachi H‐7500, Japan) at 80 kV.

#### 2.3.3 Immunofluorescence (IF) Staining

Mouse brain tissues fixed with 4% PFA were dehydrated through a graded ethanol series, embedded in paraffin, and sectioned into 20 μm‐thick serial sections. After heat‐mediated antigen retrieval in ethylenediaminetetraacetic acid (EDTA) antigen retrieval buffer (pH 8.0), treatment with an autofluorescence quenching reagent, and blocking, the sections were incubated with primary antibodies overnight at 4°C. On the following day, after washing with PBS, the sections were incubated with the corresponding secondary antibodies for 1 h in the dark, followed by nuclear staining with 4′,6‐diamidino‐2‐phenylindole (DAPI; Southern Biotech, USA) for 2 min. After mounting with ProLong Diamond (Invitrogen), images were acquired using a confocal microscope (A1R‐SIM STORM, Nikon, Japan), and quantitative analysis was performed using ImageJ software (v1.47, USA). Detailed information on the primary and secondary antibodies is provided in Table S1.

### 2.4 Enzyme‐Linked Immunosorbent Assay (ELISA) of HDACs

HDAC levels in mouse hippocampal tissue were measured using an ELISA kit (Jiangsu Meimian Industrial Co. Ltd., China). Briefly, 50 mg of hippocampal tissue was homogenized in 450 μL of PBS, and the homogenate was centrifuged at 5000 rpm for 15 min at 4°C. The supernatant was then collected. Blank wells, standard wells, and sample wells were prepared according to the manufacturer's instructions. Samples were diluted fivefold before loading and incubated at 37°C for 30 min. After washing, enzyme conjugate was added, and the reaction was continued under the same conditions. Subsequently, chromogenic reagents A and B were added, and color development was allowed to proceed for 10 min at 37°C in the dark. After termination of the reaction, the absorbance at 450 nm was measured using a Tecan SunRise microplate reader. HDAC protein concentrations were calculated according to the standard curve.

### 2.5 Gas Chromatography–Mass Spectrometry (GC–MS) Analysis of SCFAs

A total of 200 μL of serum was mixed with 100 μL of 15% phosphoric acid solution and 20 μL of isohexanoic acid internal standard solution. The mixture was then extracted with 280 μL of diethyl ether and centrifuged at 12,000 rpm for 10 min at 4°C. The organic supernatant was collected for GC–MS analysis (Thermo, USA). Quantification was performed based on standard curves, and quality control (QC) samples were included to monitor instrument stability. After data preprocessing and scale normalization using the Pheatmap package in R software (v4.0.3), clustering analysis was performed.

### 2.6 Culture of HOPCs

All cells were cultured in a humidified incubator at 37°C with 5% CO_2_. Before cell seeding, culture flasks were precoated with poly‐L‐lysine (PLL, 10 mg/mL, Cat# 0413, ScienCell, USA). HOPCs were maintained in proliferation culture using Oligodendrocyte Precursor Cell Medium (OPCM), which consisted of OPCM‐b (Cat# 1601‐b, ScienCell, USA), Oligodendrocyte Precursor Cell Growth Supplement (OPCGS, Cat# 1652, ScienCell, USA), and penicillin/streptomycin solution (P/S, Cat# 0503, ScienCell, USA). After 48 h of culture, the medium was replaced with Oligodendrocyte Precursor Cell Differentiation Medium (OPCDM) to induce differentiation. OPCDM consisted of OPCM‐b, fetal bovine serum (FBS, Cat# 0500, ScienCell, USA), Oligodendrocyte Precursor Cell Differentiation Supplement (OPCDS, Cat# 1672, ScienCell, USA), and P/S. For NaB treatment, NaB was diluted to the required concentrations in complete medium and then added to the culture medium, followed by continued incubation for 24, 48, or 72 h. After treatment, the cells were collected, and the relevant indicators were analyzed using molecular biological methods.

### 2.7 Cell Counting Kit‐8 (CCK‐8) Assay

After precoating 96‐well plates with PLL, HOPCs were seeded at a density of approximately 5 × 10^3^ cells per well. When cell confluence reached approximately 70%, the cells were treated with different concentrations of NaB. Five replicate wells were set for each group, and a blank control group was included. After 24, 48, and 72 h of treatment, the culture medium containing CCK‐8 working solution (Cat# C0038, Beyotime, China) was added, and the cells were incubated at 37°C for 2 h in the dark. Cell viability was evaluated by measuring the absorbance at 450 nm using a microplate reader.

### 2.8 Flow Cytometry

HOPC proliferation was assessed using an EdU‐488 Cell Proliferation Detection Kit (Cat# C0071L‐3, Beyotime, China). After precoating 6‐well plates with PLL, HOPCs were seeded at a density of approximately 1.5 × 10^5^ cells per well. When cell confluence reached approximately 70%, the cells were treated with different concentrations of NaB for 24 h. A negative control group was included, and each group contained three replicate wells. Subsequently, 5‐ethynyl‐2′‐deoxyuridine (EdU) diluted at 1:500 was added, and the cells were incubated at 37°C for 2 h. The cells were then washed with PBS, digested, and resuspended, followed by fixation with 4% PFA, washing, permeabilization, and Click reaction. Finally, samples were analyzed by flow cytometry. Unlabeled EdU cells were used to correct for background signals, and the proportion of EdU‐positive cells was analyzed using FlowJo (v10.8.1) to calculate the proliferation rate of HOPCs.

### 2.9 Transwell Migration Assay

HOPC migratory capacity was assessed using Transwell chambers (8 μm pore size, Cat# 3422, Corning, USA). The lower chamber was filled with 500 μL of complete OPCM or complete OPCM containing 500 μM NaB, and the upper chamber was loaded with 200 μL of medium containing approximately 1 × 10^5^ HOPCs. The cells were then incubated at 37°C with 5% CO_2_ for 24 h or 48 h. After incubation, the cells were washed, fixed with 4% PFA, washed again, and stained with crystal violet (Cat# C0121, Beyotime, China) for 20 min in the dark, followed by washing. Nonmigrated cells on the upper surface of the membrane were gently removed. Three random fields were selected under a light microscope, and the number of cells on the lower surface of the membrane was counted using ImageJ software (v1.47, USA). The mean value was used to evaluate the migratory capacity of HOPCs.

### 2.10 Immunocytochemistry With Fluorescence Detection (ICC‐F)

HOPCs were seeded into confocal dishes at a density of 5 × 10^4^ cells/dish. When cell confluence reached approximately 70%, the medium was replaced with OPCDM or OPCDM containing 500 μM NaB, and the cells were incubated at 37°C for 3 days. The cells were then washed with prechilled washing buffer (Cat# C1715, Beyotime, China), fixed with 4% PFA at room temperature for 30 min, and washed again, followed by permeabilization with permeabilization buffer (Cat# P0095, Beyotime, China) for 20 min. After blocking with 5% bovine serum albumin (BSA) at room temperature for 1 h, the cells were incubated with primary antibodies overnight at 4°C. On the following day, the corresponding secondary antibodies were added and incubated for 1 h in the dark. Finally, the nuclei were stained with 1 μg/mL DAPI for 10 min. After washing, images were observed and captured under a confocal microscope.

### 2.11 Western Blot (WB)

Total protein was extracted from mouse hippocampal tissue and HOPCs using lysis buffer supplemented with phosphatase and protease inhibitors (Cat# AIWB‐012, Affinibody, China), and protein concentrations were determined using a bicinchoninic acid (BCA) protein assay kit (Cat# P0010, Beyotime, China). According to the molecular weights of the target proteins, samples were separated by sodium dodecyl sulfate‐polyacrylamide gel electrophoresis (SDS‐PAGE) and then transferred onto polyvinylidene fluoride (PVDF) membranes. After blocking with blocking buffer (Cat# AFM‐B100, Affinibody, China), the membranes were incubated with primary antibodies overnight at 4°C. On the following day, the membranes were washed with Tris‐buffered saline containing Tween‐20 (TBST) and then incubated with horseradish peroxidase (HRP)‐conjugated secondary antibodies for 1 h at room temperature. Protein bands were visualized using an enhanced chemiluminescence (ECL) reagent (Amersham Pharmacia Biotech, USA) and captured with a Chemidoc XRS imaging system (Bio‐Rad, USA). Finally, the grayscale intensity of the protein bands was quantified using ImageJ software (v1.47, USA). The original blot images are provided in Data S2, and detailed antibody information is listed in Table S2.

### 2.12 Chromatin Immunoprecipitation (ChIP)‐qPCR

Untreated HOPCs and HOPCs pretreated with 500 μM NaB were supplemented with 37% formaldehyde stock solution to a final concentration of 1% and crosslinked at room temperature for 10 min to stabilize protein‐DNA complexes. Glycine was then added to quench the crosslinking reaction, followed by incubation at room temperature for 5 min. The cells were lysed with lysis buffer containing protease inhibitors, and chromatin was fragmented to 200–800 bp using a Covaris S220 ultrasonicator. After centrifugation, the supernatant was collected, and 50 μL of chromatin solution was diluted to 500 μL with dilution buffer containing freshly added protease inhibitors. Subsequently, 2 μg of anti‐H3K9ac antibody (Cat# AB32129, Abcam, UK) or 2 μg of isotype control IgG (Cat# AB205719, Abcam, UK) was added, together with 30 μL of A/G magnetic beads, followed by overnight incubation at 4°C. The immunoprecipitated complexes were sequentially washed with low‐salt, high‐salt, lithium chloride (LiCl), and Tris‐EDTA (TE) buffers, then eluted with elution buffer and treated with Proteinase K at 65°C for 4 h to reverse crosslinking. The supernatant was subsequently collected, and DNA was purified using a QIAquick Gel Extraction Kit (Cat# 28704, Qiagen, Germany). DNA from the input samples was extracted in parallel as a quantitative reference. Finally, the enrichment of H3K9ac at the BDNF promoter region was determined by qPCR, with input DNA and IgG ChIP DNA used as negative controls. The primer sequences are listed in Table S3.

### 2.13 Reverse Transcription Quantitative Polymerase Chain Reaction (RT‐qPCR)

Total RNA was extracted from tissue and cell samples using TRIzol reagent (Ambion, USA) according to the manufacturer's instructions, and RNA concentration and purity were assessed using a NanoDrop spectrophotometer (Thermo Fisher Scientific, USA). RNA was then reverse‐transcribed into complementary deoxyribonucleic acid (cDNA) using the HiScript III RT SuperMix for qPCR (+gDNA wiper) kit (Vazyme) according to the manufacturer's protocol. Using cDNA as the template, qPCR amplification was performed with SYBR Premix Ex Taq II (Takara, China) on a Bio‐Rad CFX384 real‐time PCR system. The specific primers used in this study were synthesized by Sangon Biotech (China), and their sequences are listed in Table S3. β‐actin was used as the internal reference gene, and the relative expression levels of target genes were calculated using the 2^−ΔΔCt^ method.

### 2.14 Statistical Analysis

All statistical analyses were performed using IBM SPSS Statistics 24.0 (IBM Corp., USA) and GraphPad Prism 8 (GraphPad Software, USA). All experiments were independently repeated three times. The normality of continuous variables was assessed using the Shapiro–Wilk test. Data with a normal distribution are presented as mean ± standard deviation (mean ± SD), whereas non‐normally distributed data are presented as median and interquartile range [median (IQR)]. For comparisons between two groups, an independent‐samples *t*‐test was used when the data were normally distributed with equal variances; Welch's *t*‐test was used when the data were normally distributed but variances were unequal; and the Mann–Whitney *U* test was used when the data were not normally distributed. For comparisons among three or more groups, one‐way analysis of variance (ANOVA) was used when the data were normally distributed with equal variances; Welch's ANOVA was used when the data were normally distributed but variances were unequal; and the Kruskal‐Wallis *H* test was used when the data were not normally distributed. When two independent variables were involved, two‐way ANOVA was used to evaluate the main effects of each factor and their interaction. Post hoc multiple‐comparison analyses were performed when necessary. A *p* value < 0.05 was considered statistically significant.

## 3 Results

### 3.1 Repeated Sevoflurane Exposure Induced Neurobehavioral Impairments in Neonatal Mice

**FIGURE 1:**
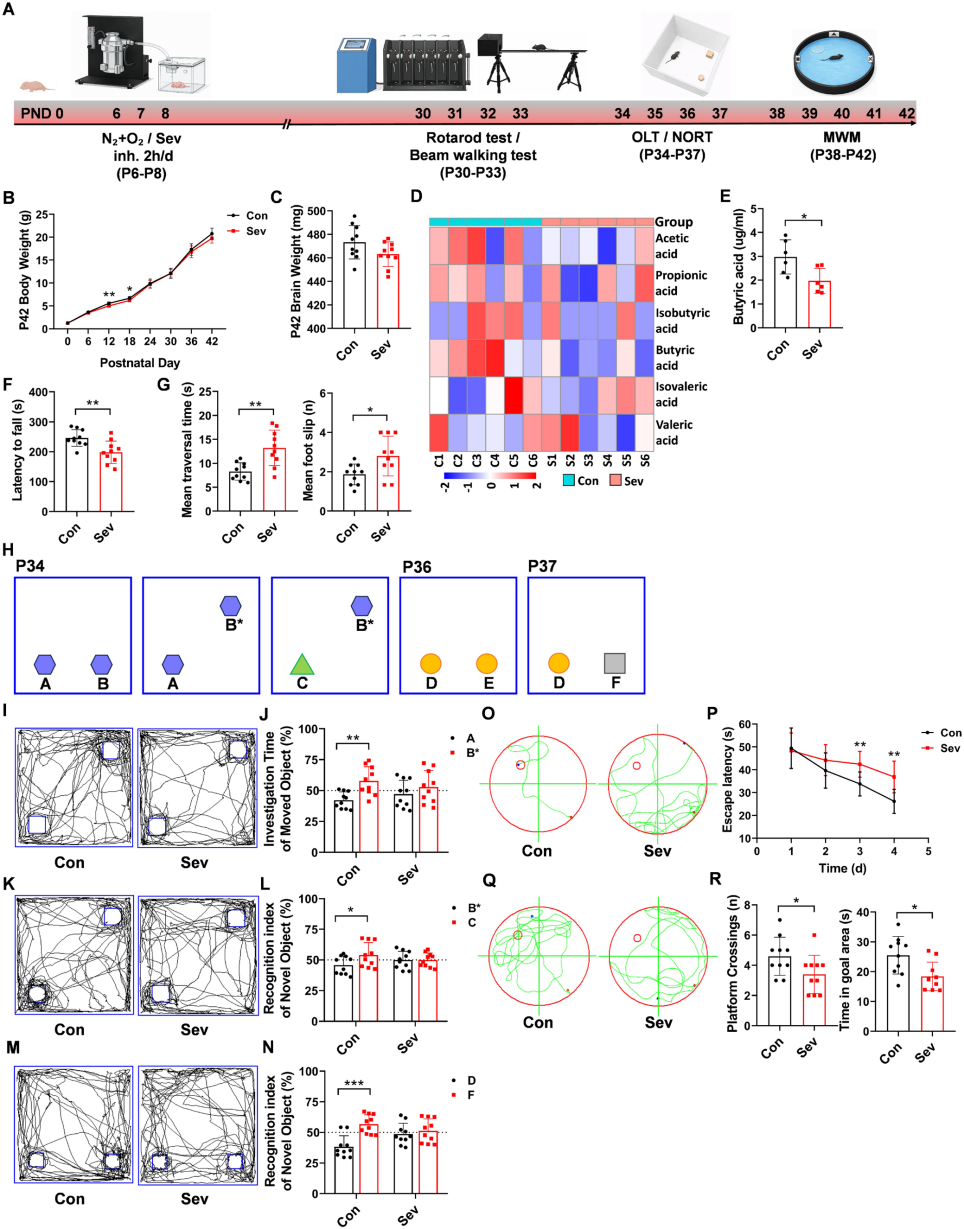
Effects of repeated sevoflurane exposure on development and neurobehavioral function in neonatal mice. (A) Experimental design: neonatal mice were exposed to sevoflurane or carrier gas for 2 h/day on P6–P8 and subjected to behavioral testing on P30–P42. (B) Body weight and (C) brain weight of mice; *n* = 10 mice/group. (D) Clustered heatmap of serum SCFAs in mice. (E) Butyrate concentration; *n* = 6 mice/group. (F) Latency to fall from the onset of rod rotation in the rotarod test; *n* = 10 mice/group. (G) Mean traversal time from the starting point to the endpoint and mean number of foot slips in the beam walking test; *n* = 10 mice/group. (H) Schematic illustration of object positions and shapes in the OLT and NORT. (I) Representative movement trajectories in the OLT and (J) exploration time for the familiar‐location object A and the novel‐location object B*; *n* = 10 mice/group. (K) Representative movement trajectories in the short‐term NORT and (L) exploration time for the familiar object B* and the novel object C; *n* = 10 mice/group. (M) Representative movement trajectories in the long‐term NORT and (N) exploration time for the familiar object D and the novel object F; *n* = 10 mice/group. (O) Representative swimming trajectories during the training phase of the MWM and (P) escape latency; *n* = 10 mice/group. (Q) Representative swimming trajectories during the probe phase of the MWM and (R) platform crossing frequency and time spent in the target quadrant; *n* = 10 mice/group. Statistical differences in body weight and escape latency over time between the two groups were assessed using two‐way ANOVA. The statistical difference in platform crossing frequency between the two groups was assessed using the Mann–Whitney *U* test. Statistical differences in all other variables between the two groups were assessed using the independent‐samples *t*‐test. A *p* value < 0.05 was considered statistically significant. Significance levels are indicated as follows: *p < 0.05, **p < 0.01, ***p < 0.001, and ****p < 0.0001.

Neonatal mice were exposed to sevoflurane on P6‐P8, and behavioral assessments were performed on P30‐P42 to evaluate neurobehavioral function (Figure 1A). Following sevoflurane exposure, the mice showed a significant trend toward reduced body weight at P12 and P18 (*F* = 9.406, *p* = 0.0066), which gradually recovered over the long term, suggesting that anesthetic exposure may exert a transient adverse effect on growth and development (Figure 1B). Brain weight was measured at P42, and no significant difference was observed, indicating that the early growth inhibition and potential neurotoxicity induced by sevoflurane did not result in a permanent reduction in overall brain size or gross structural loss (Figure 1C). Serum SCFA levels were also measured at P42 (Figure 1D). The results showed that the levels of acetate, isobutyrate, and butyrate in the sevoflurane‐exposed group all exhibited a downward trend (Figure S1), whereas only the reduction in butyrate reached statistical significance (*t* = 2.770, *p* = 0.0198) (Figure 1E). Rotarod and beam walking tests were performed on P30‐P33. As shown in Figure 1F, the sevoflurane‐exposed group exhibited a significantly shorter latency to fall in the rotarod test than the control group (*t* = 3.222, *p* = 0.0047), indicating impaired motor coordination. As shown in Figure 1G, the sevoflurane‐exposed group showed a significantly longer beam traversal time in the beam walking test than the control group (*t* = 3.787, *p* = 0.0013), along with a markedly increased number of foot slips (*t* = 2.595, *p* = 0.0183), suggesting impaired balance and fine motor control. OLT and NORT were performed on P34‐P37, and the experimental scheme is shown in Figure 1H. Figure 1I shows the representative movement trajectories of mice during the OLT. In the control group, the exploration time for the object in the novel location (B*) was significantly longer than that for the object in the familiar location (A) (*t* = 3.693, *p* = 0.0017), whereas no significant difference in exploration time between the two objects was observed in the sevoflurane‐exposed group (Figure 1J), suggesting that sevoflurane exposure impaired spatial memory. Figure 1K shows the representative movement trajectories of mice in the short‐term memory phase of the NORT. The control group spent significantly more time exploring the novel object C (*t* = 2.104, *p* = 0.0497), whereas no significant difference was found between the two objects in the sevoflurane‐exposed group (Figure 1L). Figure 1M shows the representative movement trajectories of mice in the long‐term memory phase of the NORT. The control group spent significantly more time exploring the novel object F (*t* = 4.925, *p* = 0.0001), whereas no significant difference was observed between the two objects in the sevoflurane‐exposed group (Figure 1N). These findings indicate that sevoflurane exposure impaired both short‐term and long‐term memory. Figure 1O shows the representative movement trajectories of mice during the training phase of the MWM test. On training days 3 and 4, the sevoflurane‐exposed group exhibited longer escape latency than the control group (*F* = 4.609, *p* = 0.0477) (Figure 1P), indicating impaired learning ability after sevoflurane exposure. Figure 1Q shows the representative movement trajectories of mice during the probe phase of the MWM test. We found that sevoflurane exposure impaired spatial working memory and reference memory, as evidenced by a significantly lower platform crossing frequency (*U* = 26, *p* = 0.046) and a shorter time spent in the target quadrant (*t* = 2.838, *p* = 0.0109) in the sevoflurane‐exposed group than in the control group (Figure 1R). Taken together, these results indicate that repeated sevoflurane exposure in neonatal mice leads to a series of neurobehavioral impairments.

### 3.2 Repeated Sevoflurane Exposure Inhibited OPC Proliferation and Differentiation and Impaired Myelination in Neonatal Mice

**FIGURE 2:**
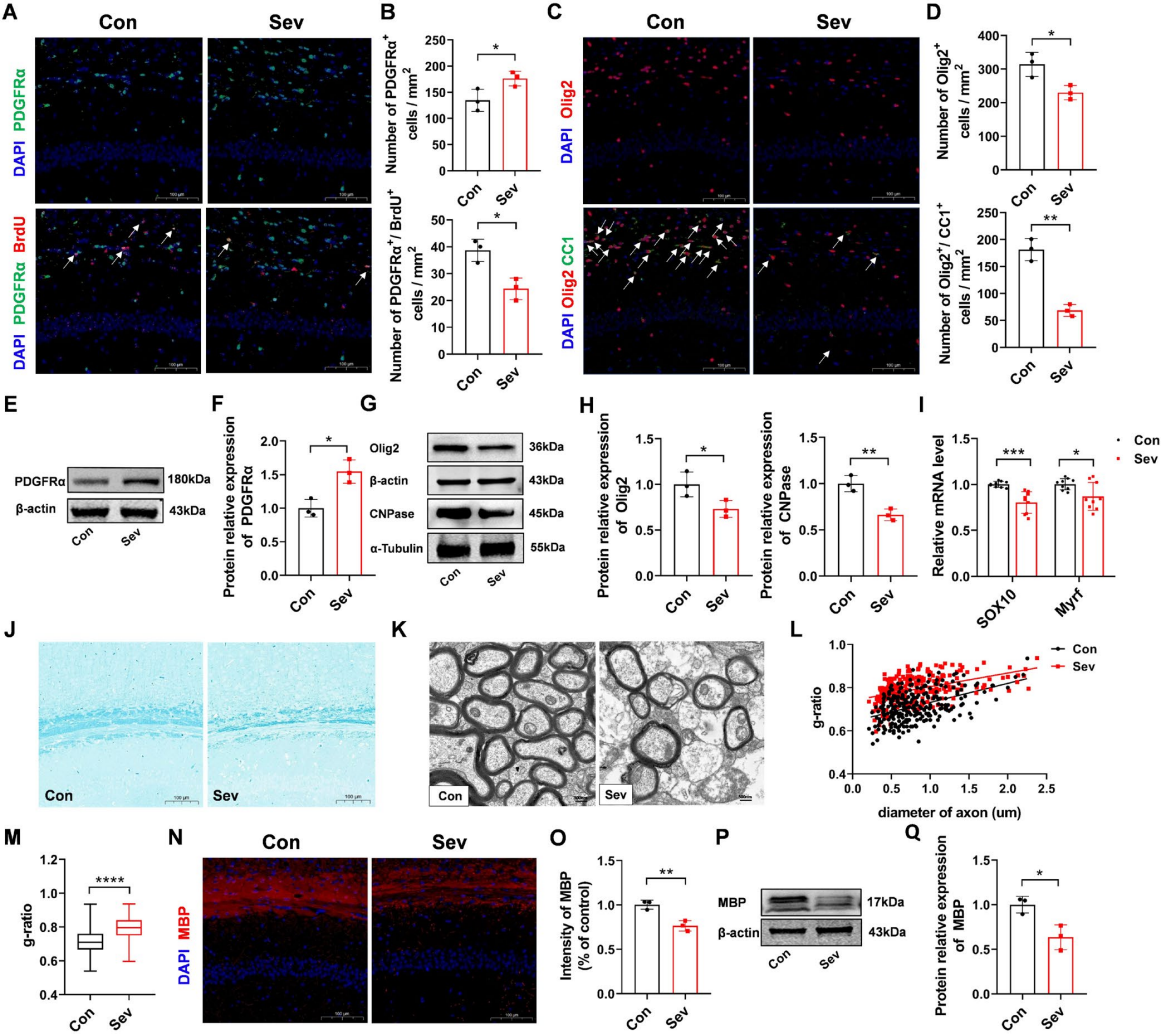
Effects of repeated sevoflurane exposure on OPC proliferation, differentiation, and myelination in neonatal mice. (A) IF staining of PDGFRα (green) and BrdU (red) in the hippocampal CA1 region. Colocalized cells (yellow) are indicated by white arrows, and nuclei were counterstained with DAPI (blue) (scale bar = 100 μm). (B) Quantitative analysis of PDGFRα^+^ cells and PDGFRα^+^/BrdU^+^ double‐positive cells; *n* = 3 mice/group. (C) IF staining of Olig2 (red) and CC1 (green) in the hippocampal CA1 region. Colocalized cells (yellow) are indicated by white arrows, and nuclei were counterstained with DAPI (blue) (scale bar = 100 μm). (D) Quantitative analysis of Olig2^+^ cells and Olig2^+^/CC1^+^ double‐positive cells; *n* = 3 mice/group. (E) WB analysis of PDGFRα protein expression in hippocampal tissue. (F) Quantitative analysis of PDGFRα protein band intensity; *n* = 3 mice/group. (G) WB analysis of Olig2 and CNPase protein expression in hippocampal tissue. (H) Quantitative analysis of Olig2 and CNPase protein band intensity; *n* = 3 mice/group. (I) RT‐qPCR analysis of SOX10 and Myrf mRNA expression levels in hippocampal tissue; *n* = 3 mice/group. (J) LFB staining showing myelin structure (scale bar = 100 μm). (K) Ultrastructure of myelin observed by TEM (scale bar = 200 nm). (L) Distribution of myelin g‐ratio across different axonal diameters. (M) Quantitative analysis of myelin g‐ratio. (N) IF staining of MBP (red) in the hippocampal CA1 region, with nuclei counterstained with DAPI (blue) (scale bar = 100 μm). (O) Quantitative analysis of MBP fluorescence intensity; *n* = 3 mice/group. (P) WB analysis of MBP protein expression in hippocampal tissue. (Q) Quantitative analysis of MBP protein band intensity; *n* = 3 mice/group. Statistical differences between the two groups were assessed using the independent‐samples *t*‐test. A *p* value < 0.05 was considered statistically significant. Significance levels are indicated as follows: *p < 0.05, **p < 0.01, ***p < 0.001, and ****p < 0.0001.

IF staining showed that repeated sevoflurane exposure increased the number of platelet‐derived growth factor receptor alpha (PDGFRα)‐positive cells (*t* = 2.856, *p* = 0.0461), suggesting that the differentiation process of OPCs was inhibited. Meanwhile, the number of BrdU/PDGFRα double‐positive cells was reduced (*t* = 4.279, *p* = 0.0129), indicating that OPC proliferation was also suppressed (Figure 2A,B). In addition, the number of oligodendrocyte transcription factor 2 (Olig2)‐positive cells was decreased (*t* = 3.511, *p* = 0.0246), and the number of adenomatous polyposis coli (APC; clone CC1)/Olig2 double‐positive cells was also reduced (*t* = 8.369, *p* = 0.0011), further confirming the inhibitory effect of sevoflurane on OPC differentiation (Figure 2C,D). Consistent with the IF findings, WB analysis showed that PDGFRα protein levels were increased in the sevoflurane group (*t* = 4.352, *p* = 0.0121) (Figure 2E,F), whereas the expression levels of Olig2 and 2′, 3′‐cyclic nucleotide 3′‐phosphodiesterase (CNPase) were both significantly decreased (*t* = 2.820, *p* = 0.0478; *t* = 5.434, *p* = 0.0056) (Figure 2G,H). SRY‐box transcription factor 10 (SOX10) and myelin regulatory factor (Myrf) are key transcription factors regulating OPC differentiation, and sevoflurane reduced the mRNA expression levels of SOX10 and Myrf in the mouse brain (*t* = 4.882, *p* = 0.0002; *t* = 2.452, *p* = 0.0260) (Figure 2I). LFB staining showed that nerve fibers in the corpus callosum of the sevoflurane‐exposed group were sparse, discontinuous, and thinner, indicating myelin damage (Figure 2J). To further evaluate myelin alterations at the ultrastructural level, TEM analysis was performed (Figure 2K). Quantitative analysis showed that the g‐ratio, indicating a thinner myelin sheath, was significantly increased in the sevoflurane group compared with the control group (*t* = 13.42, *p* < 0.0001) (Figure 2L,M), suggesting reduced myelin thickness. In addition, sevoflurane exposure also downregulated the expression of myelin basic protein (MBP) (*t* = 5.335, *p* = 0.0059; *t* = 3.781, *p* = 0.0194) (Figure 2N–Q). Taken together, these results indicate that repeated sevoflurane exposure in neonatal mice exerts long‐term adverse effects on neurodevelopment. By inhibiting OPC proliferation and differentiation and downregulating key transcription factors, sevoflurane further affects the expression of myelin structural proteins, thereby impairing normal myelin development and structural integrity. This may represent one of the major mechanisms underlying sevoflurane‐induced neurobehavioral deficits.

### 3.3 ANA‐12 Attenuated the Protective Effects of NaB Against Neurobehavioral Impairments Induced by Repeated Sevoflurane Exposure in Neonatal Mice

**FIGURE 3:**
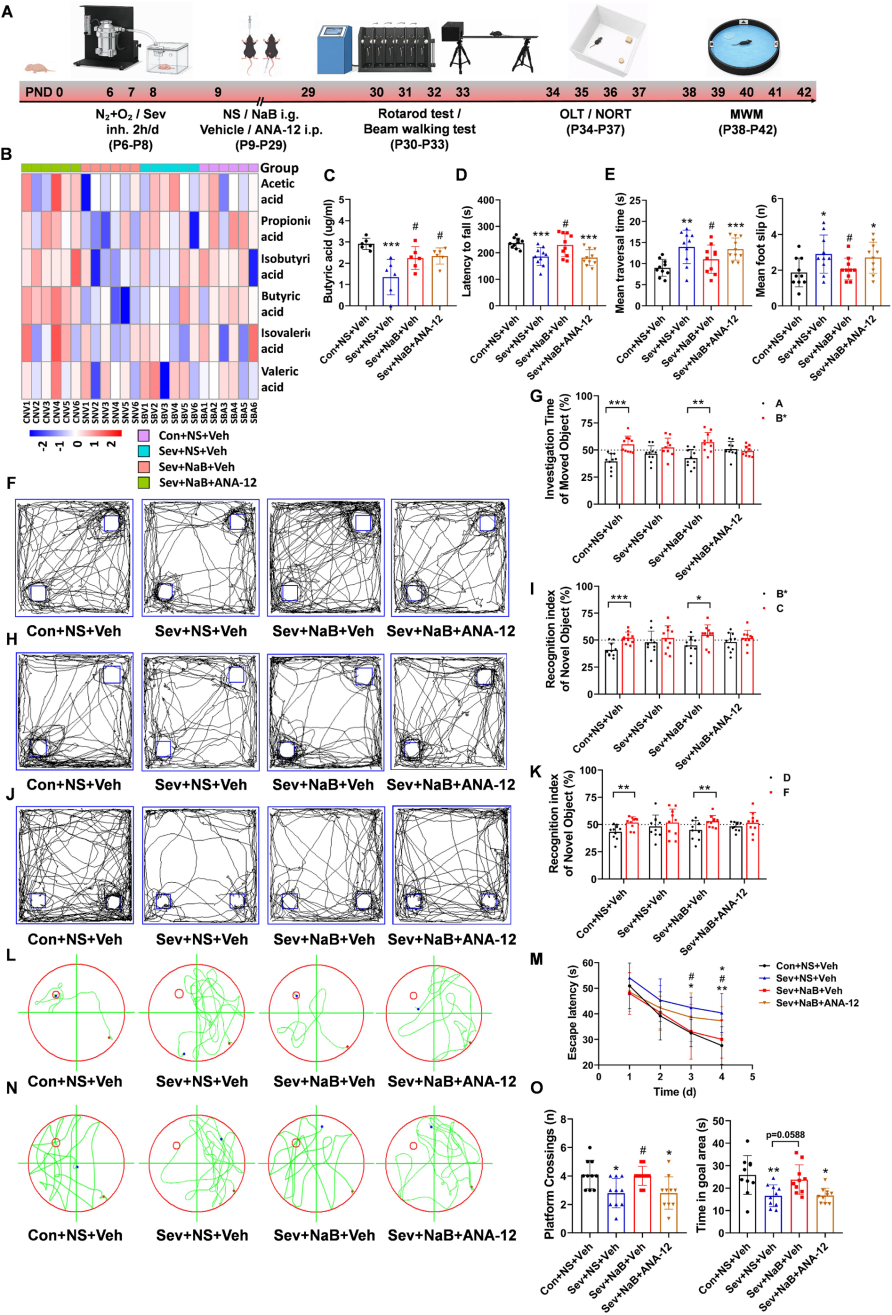
NaB ameliorated neurobehavioral impairments induced by repeated sevoflurane exposure in neonatal mice, and this effect was antagonized by ANA‐12. (A) Experimental design: neonatal mice were exposed to sevoflurane or nitrogen‐oxygen mixed gas for 2 h/day on P6–P8; from P9 to P29, mice received NaB or NS by oral gavage, together with intraperitoneal injection of ANA‐12 or vehicle; behavioral tests were performed on P30–P42. (B) Clustered heatmap of serum SCFAs in mice. (C) Butyrate concentration; *n* = 6 mice/group. (D) Latency to fall from the onset of rod rotation in the rotarod test; *n* = 10 mice/group. (E) Mean traversal time from the starting point to the endpoint and mean number of foot slips in the beam walking test; *n* = 10 mice/group. (F) Representative movement trajectories in the OLT and (G) exploration time for the familiar‐location object A and the novel‐location object B*; *n* = 10 mice/group. (H) Representative movement trajectories in the short‐term NORT and (I) exploration time for the familiar object B* and the novel object C; *n* = 10 mice/group. (J) Representative movement trajectories in the long‐term NORT and (K) exploration time for the familiar object D and the novel object F; *n* = 10 mice/group. (L) Representative swimming trajectories during the training phase of the MWM and (M) escape latency; *n* = 10 mice/group. (N) Representative swimming trajectories during the probe phase of the MWM and (O) platform crossing frequency and time spent in the target quadrant; *n* = 10 mice/group. Statistical differences in serum SCFA levels and time spent in the target quadrant in the MWM among multiple groups were assessed using one‐way ANOVA. Statistical differences in changes in escape latency among groups were assessed using two‐way ANOVA. Statistical differences in platform crossing frequency among groups were assessed using the Kruskal‐Wallis *H* test. Pairwise differences in all other variables among groups were assessed using the independent‐samples *t*‐test. A *p* value < 0.05 was considered statistically significant. Significance levels are indicated as follows: Compared with the Con+NS + Veh group, *p < 0.05, **p < 0.01, ***p < 0.001, and ****p < 0.0001; compared with the Sev + NS + Veh group, #*p* < 0.05, ##*p* < 0.01, ###*p* < 0.001, and ####*p* < 0.0001.

Mice received NaB by oral gavage from P9 to P29. To investigate the molecular mechanism underlying the protective effects of NaB, the TrkB antagonist ANA‐12 was co‐administered to a subset of mice during the treatment period (Figure 3A). Serum SCFA analysis at P42 showed that NaB gavage partially reversed the sevoflurane‐induced reduction in serum butyrate levels (*F* = 8.383, *p* = 0.0008) (Figure 3B,C), but had no significant effect on the levels of other SCFAs (Figure S2). Figure 3D shows that, in the rotarod test, NaB treatment markedly prolonged the latency to fall in sevoflurane‐exposed mice, indicating that NaB alleviated sevoflurane‐induced motor dysfunction. Furthermore, after co‐administration of ANA‐12, the beneficial effect of NaB on latency to fall was significantly attenuated, suggesting that ANA‐12 blocked the protective effect of NaB (*F* = 7.445, *p* = 0.0005). Figure 3E shows that, in the beam walking test, NaB treatment significantly shortened the beam traversal time and reduced the number of foot slips in sevoflurane‐exposed mice. After co‐administration of ANA‐12, these beneficial effects were markedly weakened, further indicating that ANA‐12 inhibited the protective effects of NaB against sevoflurane‐induced motor impairment (*F* = 5.549, *p* = 0.0031; *F* = 3.375, *p* = 0.0287). Figure 3F shows the representative movement trajectories of the four groups during the OLT. NaB treatment effectively restored the impairments in spatial memory and learning induced by sevoflurane exposure, whereas co‐administration of ANA‐12 significantly blocked this protective effect of NaB (*t* = 4.726, *p* = 0.0002; *t* = 3.893, *p* = 0.0011) (Figure 3G). Figure 3H,J show the representative movement trajectories of mice in the NORT. NaB treatment similarly reversed the sevoflurane‐induced impairments in short‐term and long‐term memory, and this effect was also blocked by ANA‐12 (*t* = 4.256, *p* = 0.0005; *t* = 2.481, *p* = 0.0232; *t* = 3.239, *p* = 0.0046; *t* = 2.496, *p* = 0.0225) (Figure 3I,K). Figure 3L,N show the representative movement trajectories of mice during the training phase and probe phase of the MWM. Further experiments confirmed that NaB treatment improved both learning performance during the training phase and spatial memory performance during the probe trial in sevoflurane‐exposed mice, and these improvements were attenuated by ANA‐12 (*F* = 7.332, *p* = 0.0006; *H* = 13.71, *p* = 0.003; *F* = 6.094, *p* = 0.0018) (Figure 3M,O). Taken together, these results suggest that NaB may alleviate sevoflurane‐induced neurobehavioral impairments through activation of a TrkB‐dependent signaling pathway.

### 3.4 ANA‐12 Attenuated the Protective Effects of NaB Against Sevoflurane‐Induced Impairments in OPC Proliferation and Differentiation and Defective Myelination in Neonatal Mice

**FIGURE 4:**
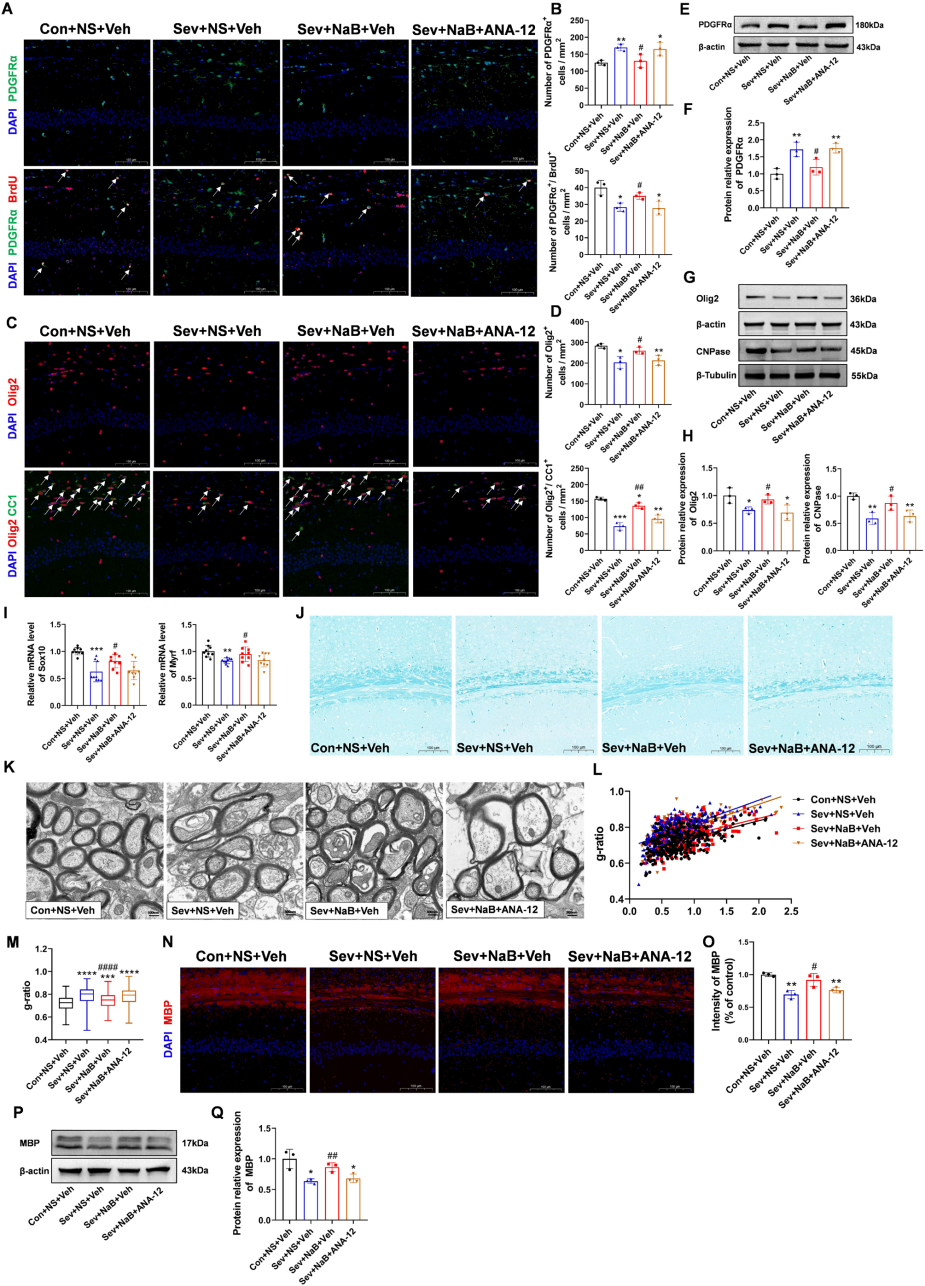
Mechanisms underlying the protective effects of NaB against sevoflurane‐induced impairments in OPC proliferation, differentiation, and myelination in neonatal mice, and the antagonistic effects of ANA‐12. (A) IF staining of PDGFRα (green) and BrdU (red) in the hippocampal CA1 region. Colocalized cells (yellow) are indicated by white arrows, and nuclei were counterstained with DAPI (blue) (scale bar = 100 μm). (B) Quantitative analysis of PDGFRα^+^ cells and PDGFRα^+^/BrdU^+^ double‐positive cells; *n* = 3 mice/group. (C) IF staining of Olig2 (red) and CC1 (green) in the hippocampal CA1 region. Colocalized cells (yellow) are indicated by white arrows, and nuclei were counterstained with DAPI (blue) (scale bar = 100 μm). (D) Quantitative analysis of Olig2^+^ cells and Olig2^+^/CC1^+^ double‐positive cells; *n* = 3 mice/group. (E) WB analysis of PDGFRα protein expression in hippocampal tissue. (F) Quantitative analysis of PDGFRα protein band intensity; *n* = 3 mice/group. (G) WB analysis of Olig2 and CNPase protein expression in hippocampal tissue. (H) Quantitative analysis of Olig2 and CNPase protein band intensity; *n* = 3 mice/group. (I) RT‐qPCR analysis of SOX10 and Myrf mRNA expression levels in hippocampal tissue; *n* = 3 mice/group. (J) LFB staining showing myelin structure (scale bar = 100 μm). (K) Ultrastructure of myelin observed by TEM (scale bar = 200 nm). (L) Distribution of myelin g‐ratio across different axonal diameters. (M) Quantitative analysis of myelin g‐ratio. (N) IF staining of MBP (red) in the hippocampal CA1 region, with nuclei counterstained with DAPI (blue) (scale bar = 100 μm). (O) Quantitative analysis of MBP fluorescence intensity; *n* = 3 mice/group. (P) WB analysis of MBP protein expression in hippocampal tissue. (Q) Quantitative analysis of MBP protein band intensity; *n* = 3 mice/group. Statistical differences among multiple groups for all variables were assessed using one‐way analysis of variance (ANOVA). A *p* value < 0.05 was considered statistically significant. Significance levels are indicated as follows: Compared with the Con + NS + Veh group, *p < 0.05, **p < 0.01, ***p < 0.001, and ****p < 0.0001; compared with the Sev + NS + Veh group, #*p* < 0.05, ##*p* < 0.01, ###*p* < 0.001, and ####*p* < 0.0001.

IF staining showed that NaB treatment reduced the sevoflurane‐induced increase in the number of PDGFRα‐positive cells (*F* = 9.019, *p* = 0.0060) and increased the number of BrdU/PDGFRα double‐positive cells (*F* = 6.849, *p* = 0.0134) (Figure 4A,B). NaB treatment also restored the reduced number of Olig2‐positive cells (*F* = 9.683, *p* = 0.0049) and increased the number of CC1/Olig2 double‐positive cells (*F* = 8.486, *p* = 0.00038) (Figure 4C,D), confirming that it could alleviate the inhibitory effects of sevoflurane on OPC proliferation and differentiation. Consistent with the IF findings, WB analysis showed that NaB effectively reversed the sevoflurane‐induced increase in PDGFRα protein expression (*F* = 11.92, *p* = 0.0025) (Figure 4E,F) as well as the decreases in Olig2 and CNPase protein expression (*F* = 5.404, *p* = 0.0251; *F* = 10.15, *p* = 0.0042) (Figure 4G,H). In addition, NaB improved the sevoflurane‐induced reductions in SOX10 and Myrf mRNA expression (*F* = 8.334, *p* = 0.00051; *F* = 5.517, *p* = 0.0036) (Figure 4I), alleviated the pathological changes in the corpus callosum characterized by sparse, discontinuous, and thinned nerve fibers (Figure 4J), reduced the elevated g‐ratio (*F* = 29.49, *p* < 0.0001) (Figure 4K–M), and restored the downregulated expression of MBP (*F* = 14.8, *p* = 0.0013; *F* = 9.388, *p* = 0.0053) (Figure 4N–Q). However, all of these protective effects were abolished by ANA‐12. Taken together, these findings indicate that NaB markedly ameliorates sevoflurane‐induced impairments in OPC proliferation and differentiation as well as myelination deficits, at least in part through activation of the BDNF/TrkB signaling pathway.

### 3.5 NaB Restored the Reductions in BDNF, p‐TrkB, and Histone Acetylation Levels in the Hippocampus of Neonatal Mice Induced by Repeated Sevoflurane Exposure

**FIGURE 5:**
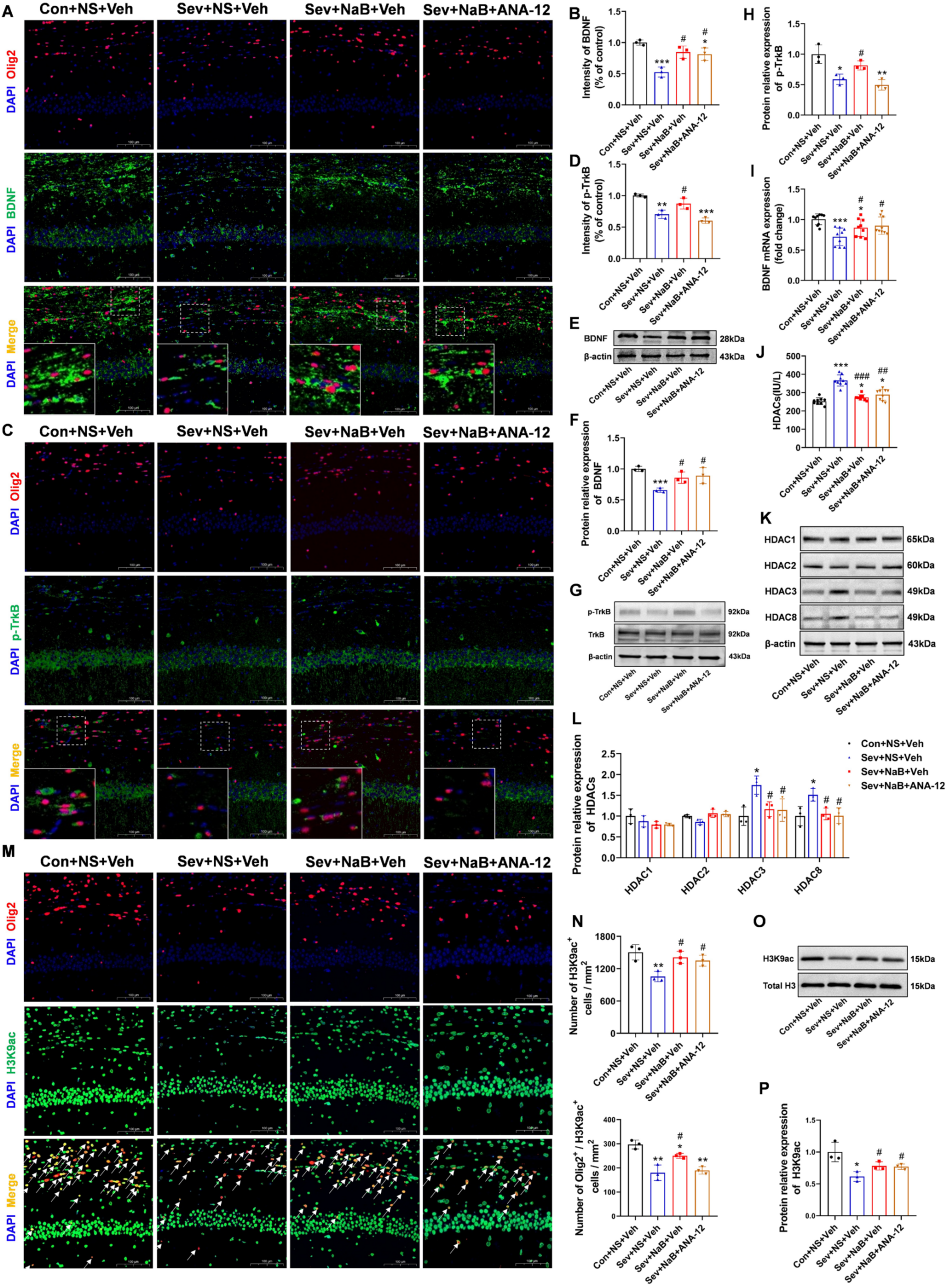
Regulatory effects of repeated sevoflurane exposure on histone acetylation and the BDNF/TrkB signaling pathway in neonatal mice, and the ameliorative effects of NaB. (A) IF staining of BDNF (green) and Olig2 (red) in the hippocampal CA1 region, with nuclei counterstained with DAPI (blue) (scale bar = 100 μm). (B) Quantitative analysis of BDNF fluorescence intensity; *n* = 3 mice/group. (C) IF staining of p‐TrkB (green) and Olig2 (red) in the hippocampal CA1 region, with nuclei counterstained with DAPI (blue) (scale bar = 100 μm). (D) Quantitative analysis of p‐TrkB fluorescence intensity; *n* = 3 mice/group. (E) WB analysis of BDNF protein expression in hippocampal tissue. (F) Quantitative analysis of BDNF protein band intensity; *n* = 3 mice/group. (G) WB analysis of p‐TrkB and TrkB protein expression in hippocampal tissue. (H) Quantitative analysis of p‐TrkB and TrkB protein band intensity; *n* = 3 mice/group. (I) RT‐qPCR analysis of BDNF mRNA expression levels in hippocampal tissue; *n* = 3 mice/group. (J) ELISA analysis of HDAC levels in hippocampal tissue; *n* = 3 mice/group. (K) WB analysis of HDAC1, HDAC2, HDAC3, and HDAC8 protein expression in hippocampal tissue. (L) Quantitative analysis of HDAC1, HDAC2, HDAC3, and HDAC8 protein band intensity; *n* = 3 mice/group. (M) IF staining of H3K9ac (green) and Olig2 (red) in the hippocampal CA1 region. Colocalized cells (yellow) are indicated by white arrows, and nuclei were counterstained with DAPI (blue) (scale bar = 100 μm). (N) Quantitative analysis of H3K9ac^+^ cells and H3K9ac^+^/Olig2^+^ double‐positive cells; *n* = 3 mice/group. (O) WB analysis of H3K9ac protein expression in hippocampal tissue. (P) Quantitative analysis of H3K9ac protein band intensity; *n* = 3 mice/group. Statistical differences among multiple groups for all variables were assessed using one‐way ANOVA. A *p* value < 0.05 was considered statistically significant. Significance levels are indicated as follows: Compared with the Con+NS + Veh group, *p < 0.05, **p < 0.01, ***p < 0.001, and ****p < 0.0001; compared with the Sev + NS + Veh group, #*p* < 0.05, ##*p* < 0.01, ###*p* < 0.001, and ####*p* < 0.0001.

Next, we investigated the specific molecular mechanisms by which NaB ameliorates sevoflurane‐induced neurobehavioral impairments. Previous studies have shown that sevoflurane disrupts maternal lipid metabolism through the BDNF/TrkB signaling pathway and impairs learning and memory in offspring rats [15]. Butyrate has also been reported to reverse the reduction in BDNF levels induced by stress and obesity models, accompanied by improvement in cognitive behavior [16, 17]. In our study, repeated sevoflurane exposure in neonatal mice reduced BDNF expression in the hippocampus compared with that in control mice, and NaB treatment restored this change (*F* = 16.58, *p* = 0.0009) (Figure 5A,B). On this basis, we further examined the expression of p‐TrkB to assess the activation status of the BDNF/TrkB signaling pathway. IF staining showed that sevoflurane exposure decreased p‐TrkB expression in the hippocampus, whereas NaB treatment restored this change (*F* = 19.39, *p* = 0.0002) (Figure 5C,D). Consistent with the IF results, WB analysis showed that NaB treatment reversed the sevoflurane‐induced reductions in BDNF protein levels (*F* = 8.761, *p* = 0.0066) and p‐TrkB protein levels (*F* = 14.21, *p* = 0.0014) (Figure 5E–H). NaB treatment also restored the sevoflurane‐induced decrease in BDNF mRNA expression (*F* = 7.203, *p* = 0.0008) (Figure 5I). To further explore the potential epigenetic mechanism by which NaB restores BDNF expression and activates the TrkB receptor, we measured HDAC levels. The results showed that sevoflurane increased HDAC levels in the hippocampus, whereas NaB treatment reversed this change (*F* = 27.25, *p* < 0.0001) (Figure 5J). Previous studies have shown that NaB primarily inhibits class I HDACs, followed by class IIa HDACs, but has no significant effect on class IIb, III, or IV HDACs [18]. Accordingly, we examined the class I HDAC isoforms and found that sevoflurane increased the protein levels of HDAC3 and HDAC8, both of which were reduced by NaB treatment (*F* = 6.402, *p* = 0.0161; *F* = 5.527, *p* = 0.0237) (Figure 5K,L). H3K9ac is a classical acetylation site closely associated with transcriptional activation, and its alteration is strongly linked to the inhibition of HDAC activity [19]. Sevoflurane exposure reduced the number of H3K9ac‐positive cells (*F* = 8.548, *p* = 0.0071), and the number of H3K9ac/Olig2 double‐positive cells was also significantly decreased. NaB treatment restored these changes (*F* = 11.47, *p* = 0.0031) (Figure 5M,N). This trend was further confirmed by WB analysis (*F* = 3.517, *p* = 0.0460) (Figure 5O,P).

### 3.6 NaB Upregulated H3K9ac Modification, BDNF Expression, and p‐TrkB Levels in HOPCs in a Concentration‐Dependent Manner, Increased H3K9ac Enrichment at the BDNF Promoter, and Promoted HOPC Proliferation, Migration, and Differentiation

**FIGURE 6:**
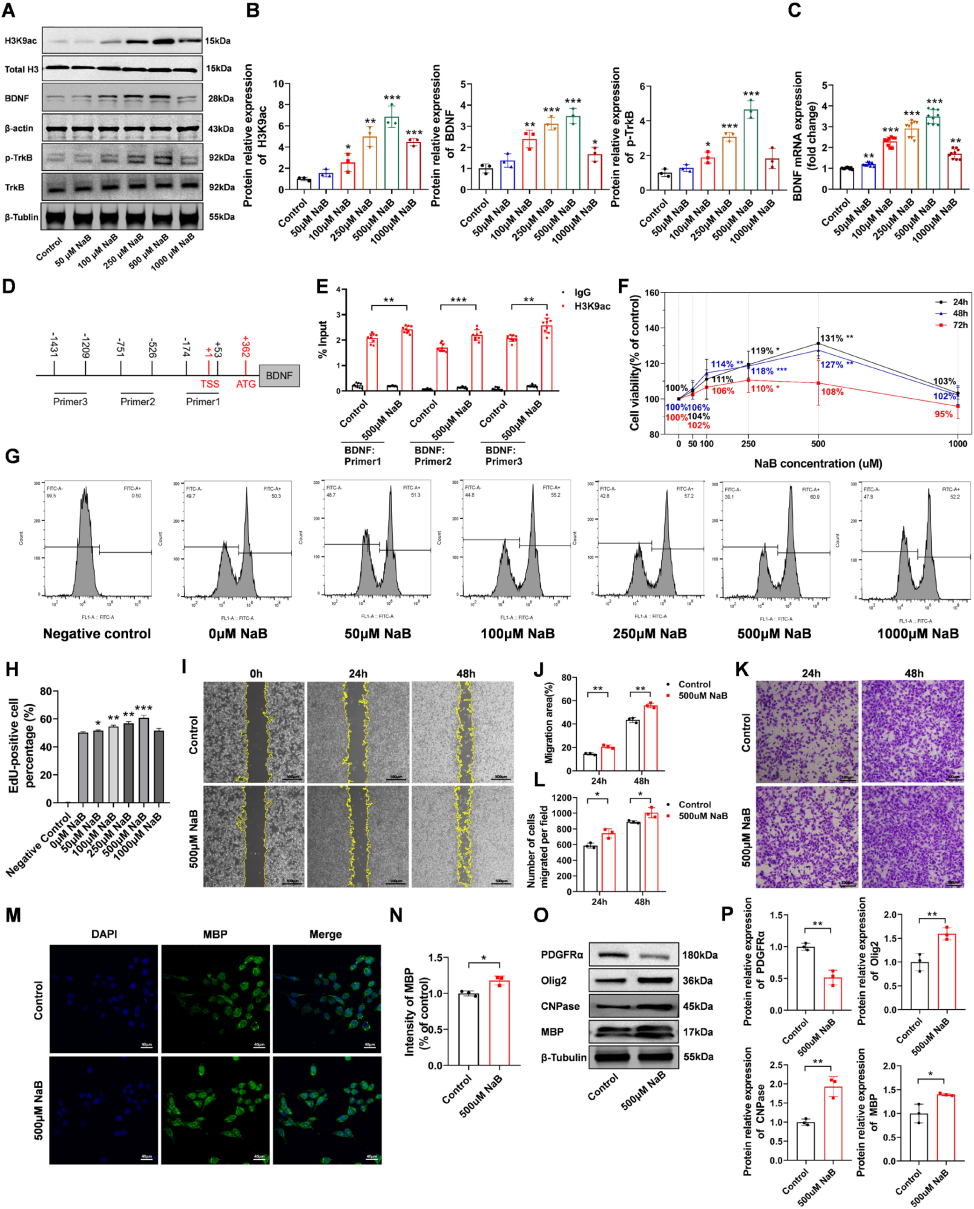
Direct effects of NaB on HOPCs. (A) WB analysis of the effects of different concentrations of NaB on H3K9ac, BDNF, and p‐TrkB protein expression in HOPCs. (B) Quantitative analysis of H3K9ac, BDNF, and p‐TrkB protein band intensity. (C) RT‐qPCR analysis of BDNF mRNA expression levels in HOPCs. (D) Schematic illustration of the BDNF promoter region. (E) ChIP‐qPCR analysis of enriched BDNF promoter fragments immunoprecipitated using anti‐H3K9ac antibody or IgG antibody. (F) CCK‐8 assay showing HOPC viability. (G) EdU proliferation analysis by flow cytometry. (H) Statistical analysis of EdU proliferation data. (I) Representative images from the scratch assay (scale bar = 500 μm). (J) Quantitative analysis of cell migratory capacity based on changes in scratch area over time. (K) Representative images from the Transwell assay (scale bar = 100 μm). (L) Quantitative analysis of cell migratory capacity based on the number of cells that successfully migrated through the Transwell membrane in each microscopic field. (M) IF staining of MBP (green) after 3 days of differentiation of HOPCs, with nuclei counterstained with DAPI (blue) (scale bar = 40 μm). (N) Quantitative analysis of MBP fluorescence intensity. (O) WB analysis of PDGFRα, Olig2, CNPase, and MBP protein expression after 3 days of HOPC differentiation. (P) Quantitative analysis of PDGFRα, Olig2, CNPase, and MBP protein band intensity. Statistical differences among multiple groups were assessed using one‐way analysis of variance (ANOVA), whereas statistical differences between two groups were assessed using the independent‐samples *t*‐test. A *p* value < 0.05 was considered statistically significant. Significance levels are indicated as follows: *p < 0.05, **p < 0.01, ***p < 0.001, and ****p < 0.0001.

To further clarify the direct role of butyrate as a key mediator of sevoflurane‐induced neurotoxicity, HOPCs were directly treated with different concentrations of NaB. WB analysis showed that NaB concentration‐dependently increased H3K9ac levels, BDNF protein expression, and p‐TrkB protein levels in HOPCs. Compared with the control group, low concentrations of NaB (≤ 50 μM) had no significant effect on H3K9ac levels, BDNF protein expression, or p‐TrkB protein levels, whereas NaB at 100–500 μM significantly increased the expression of these proteins. Notably, at higher concentrations (≥ 1000 μM), the extent of these increases was attenuated (*F* = 13.12, *p* = 0.0005; *F* = 17.36, *p* = 0.0003; *F* = 19.14, *p* = 0.0002) (Figure 6A,B). RT‐qPCR analysis showed that NaB also increased BDNF mRNA expression in HOPCs in a concentration‐dependent manner (*F* = 14.14, *p* = 0.0006) (Figure 6C). Figure 6D illustrates the location of the BDNF promoter. ChIP‐qPCR results showed that H3K9ac indeed bound to the BDNF promoter region, and the binding enrichment differed among primer sites. This finding directly confirmed that NaB promotes the binding of H3K9ac to the BDNF promoter by increasing H3K9ac modification, thereby regulating the transcriptional activation of the BDNF gene (*t* = 3.535, *p* = 0.0030; *t* = 4.765, p = 0.0002; *t* = 3.818, *p* = 0.0026) (Figure 6E). We then investigated the effects of NaB on the proliferative capacity of HOPCs. Figure 6F shows the effects of different concentrations of NaB on HOPC viability after 24, 48, and 72 h of treatment. The results showed that the effects of NaB on HOPC viability were both concentration‐ and time‐dependent. Low‐to‐moderate concentrations (≤ 500 μM) transiently promoted cell viability, whereas high‐concentration treatment (1000 μM) or prolonged exposure (72 h) reduced cell viability to levels comparable to or slightly below those of the control group. Flow cytometry further showed that NaB promoted HOPC proliferation in a concentration‐dependent manner, with 500 μM NaB producing the most pronounced pro‐proliferative effect. Specifically, the proportion of FITC‐A‐positive cells reached 60.9%, representing a 10.6% increase compared with the 0 μM NaB group. In contrast, high‐concentration treatment with 1000 μM NaB weakened this proliferative effect, with the proportion of FITC‐A‐positive cells decreasing to 52.2%, further confirming that excessively high concentrations of NaB may adversely affect HOPC proliferation (*F* = 18.53, *p* = 0.0002) (Figure 6G,H). We next examined the effects of NaB on HOPC migration. In the scratch assay, the images showed that the scratch area gradually narrowed over time in both the control group and the 500 μM NaB‐treated group, indicating that HOPCs possessed migratory capacity. Compared with the control group, the 500 μM NaB‐treated group exhibited a significantly faster wound‐healing rate (*t* = 6.105, *p* = 0.0036; *t* = 7.255, *p* = 0.0019) (Figure 6I,J). Figure 6K shows that the density of cells on the lower side of the Transwell chamber gradually increased over time, and the number of cells in the 500 μM NaB‐treated group was markedly greater than that in the control group (*t* = 4.050, *p* = 0.0155; *t* = 3.022, *p* = 0.0391) (Figure 6L), indicating that NaB effectively promoted HOPC migration. Finally, we examined the effects of NaB on HOPC differentiation. After HOPCs were cultured in differentiation medium for 3 days, IF staining showed that NaB significantly upregulated MBP protein expression (*t* = 4.424, *p* = 0.0115) (Figure 6M,N). WB analysis further confirmed this pro‐differentiation effect (*t* = 6.546, *p* = 0.0028; *t* = 4.658, *p* = 0.0096; *t* = 5.918, *p* = 0.0041; *t* = 3.469, *p* = 0.0256) (Figure 6O,P). Together, these results indicate that NaB effectively promotes the differentiation of HOPCs toward mature OLs.

## 4 Discussion

Sevoflurane has become a commonly used anesthetic for infant surgery because of its rapid onset, controllable anesthetic depth, and favorable safety profile [19]. Although extensive studies have investigated sevoflurane exposure‐induced neurobehavioral injury, there is still a lack of specific therapeutic strategies for sevoflurane‐induced neurotoxicity [20]. In the present study, repeated sevoflurane exposure in neonatal mice led to persistent memory and cognitive deficits as well as fine motor impairment in adulthood, accompanied by suppressed OPC proliferation and differentiation, abnormal myelination, reduced hippocampal BDNF expression, inhibited TrkB phosphorylation, and decreased H3K9ac levels. More importantly, NaB intervention significantly ameliorated these abnormalities, whereas the TrkB antagonist ANA‐12 markedly attenuated this protective effect, suggesting that the neuroprotective effects of NaB are at least partly dependent on activation of the BDNF/TrkB signaling pathway. Overall, this study reveals a potential novel mechanism underlying sevoflurane‐related neurodevelopmental injury through the following cascade: altered gut metabolites, epigenetic dysregulation, impaired neurotrophic signaling, OPC dysfunction, and abnormal myelination.

In our previous study, we found that sevoflurane exposure in mice induced long‐term gut microbiota dysbiosis and reduced intestinal SCFAs levels to varying degrees [8]. Butyrate, an SCFA produced by beneficial gut bacteria during food fermentation, not only exerts positive effects on intestinal development, microbial homeostasis, and host immunity [21], but also positively regulates the CNS [22]. Butyrate is closely associated with neurological disorders. It can reduce β‐amyloid deposition, attenuate neuroinflammation, and exert neuroprotective effects in models of Alzheimer's disease (AD) [23]. Recent studies have further revealed that butyrate levels are closely associated with clinical severity in patients with Parkinson's disease, and that modulation of butyrate levels can significantly improve motor symptoms [24]. Al Borhan et al. [25] reported that butyrate significantly ameliorates apoptosis‐regulatory proteins and reverses the phosphorylation of extracellular signal‐regulated kinase and p38, indicating its potential therapeutic value in neurodegenerative diseases. In the present study, we further found that sevoflurane exposure significantly reduced serum butyrate levels in mice and was accompanied by behavioral abnormalities and impaired myelination, whereas exogenous NaB supplementation partially reversed these changes. These findings suggest that butyrate deficiency may not be merely an accompanying phenomenon, but rather an important intermediary linking sevoflurane exposure to abnormal neurodevelopment. In other words, the neurotoxic effects of sevoflurane may arise not only from its direct actions on brain tissue, but also from indirect amplification through disruption of the gut metabolic environment.

Myelin is a multilamellar lipid‐rich insulating membrane structure that ensheathes neuronal axons. Through electrical insulation and saltatory conduction, it markedly enhances the speed and efficiency of nerve impulse transmission while maintaining the precise propagation of neural signals, thereby providing the structural basis for normal nervous system development and functional maintenance [26]. Normal proliferation, migration, and differentiation of OPCs are fundamental to myelination, and early life represents a period during which this process is highly active and particularly vulnerable to external disturbances [27]. Zhang et al. [28] reported that sevoflurane induces folate metabolism disorders in juvenile rhesus monkeys and mice, thereby leading to myelination defects in the developing brain. Recent studies have further shown that sevoflurane exposure in neonatal mice inhibits zinc finger DHHC‐type palmitoyltransferase 5 (ZDHHC5)‐mediated palmitoylation of transferrin receptor 1 (TfR1) in OLs, resulting in dysmyelination and neurological dysfunction [29]. These findings are consistent with our results, showing that anesthetic exposure suppresses OPC proliferation and differentiation, reduces the expression of key proteins involved in OL development, differentiation, and myelin formation, and consequently leads to defective myelination. Notably, NaB intervention significantly enhanced OPC proliferation, partially restored deficits in differentiation and maturation, and improved behavioral outcomes, thereby providing a novel potential therapeutic strategy for sevoflurane‐induced neurotoxicity.

At the mechanistic level, the BDNF/TrkB signaling pathway may serve as a key hub linking alterations in butyrate levels to abnormal myelination. BDNF participates throughout the entire process of neurodevelopment and plays a central role in the precise establishment of nervous system structure and function by regulating neuronal survival, axonal growth and target innervation, as well as synapse formation and plasticity [30, 31]. Although BDNF is well known for its critical role in neuronal function [32], its involvement in glial cell functions such as myelination has been far less extensively studied. In a study using BDNF knockout (KO) mice, the proportion of myelinated axons in the optic nerve at P20 was reduced by approximately 50% compared with that in wild‐type littermates, accompanied by a significant reduction in axonal diameter. Notably, although myelination of the optic nerve, which belongs to the CNS, was markedly affected, myelination of the facial nerve and sciatic nerve, both of which belong to the peripheral nervous system (PNS), was not significantly disturbed [33]. These findings suggest that the effects of BDNF are to some extent region‐specific, primarily influencing myelination in the CNS while exerting little apparent effect on myelin development in the PNS. Accordingly, we speculate that sevoflurane‐induced downregulation of BDNF may represent an important upstream event contributing to myelination defects. BDNF exerts its biological effects through two receptors, namely the p75 neurotrophin receptor (p75NTR) and TrkB [34, 35]. Although p75NTR KO mice survive into adulthood, they exhibit severe sensory neuron loss and myelination defects in the PNS [36], However, there is currently no evidence that p75NTR deficiency affects myelination in the CNS. Further studies have shown that the addition of BDNF to OPC cultures derived from p75NTR KO mice still effectively promotes myelination [37], further supporting the notion that TrkB, rather than p75NTR, is the essential receptor mediating the promyelinating effects of BDNF in the CNS. However, because systemic TrkB KO mice die during the early postnatal period [38], their direct application in studies of BDNF/TrkB‐mediated regulation of CNS myelination is limited. Therefore, alternative genetic or pharmacological approaches are needed to further clarify the contribution of TrkB activation in OLs to myelin development in vivo. In the present study, we used ANA‐12, a selective inhibitor that binds to an allosteric site of TrkB. By interfering with the binding of BDNF to TrkB, ANA‐12 inhibits TrkB autophosphorylation and downstream signaling, thereby effectively antagonizing BDNF/TrkB‐mediated neurobiological effects and helping to further define the downstream mechanism by which BDNF regulates myelination.

In addition to neurotrophic signaling, the present study further elucidated the above process from an epigenetic perspective. Histone acetylation is an important epigenetic modification in eukaryotic cells and refers to the post‐translational addition of acetyl groups to lysine residues on the N‐terminal tails of histones [39]. This process is catalyzed by HATs and reversed by HDACs, and together they constitute a core mechanism regulating gene transcription through alteration of chromatin conformation [40]. Among the various modification sites, H3K9ac is a classical histone mark closely associated with gene activation. It is mainly enriched in the promoter regions of actively transcribed genes, and increased acetylation at this site promotes chromatin relaxation, thereby driving transcriptional activation and enhancing gene expression [41]. Given that butyrate is a natural HDAC inhibitor, we further investigated whether butyrate mediates myelination and neurobehavioral impairment by increasing H3K9ac levels, transcriptionally activating BDNF, and thereby regulating the BDNF/TrkB signaling pathway. Previous studies have shown that NaB primarily inhibits class I HDACs, followed by class IIa HDACs, but has no significant effect on class IIb, III, or IV HDACs [18]. Based on this mechanistic hypothesis, we focused preferentially on the classical Zn^2+^‐dependent HDACs that are more closely involved in nuclear histone deacetylation and transcriptional regulation, particularly class I HDACs. Our results showed that sevoflurane exposure significantly upregulated HDAC3 and HDAC8 expression in the hippocampus, reduced H3K9ac levels in OPCs, and induced chromatin compaction, thereby suppressing the transcription of key genes, decreasing BDNF mRNA expression, and inhibiting the BDNF/TrkB signaling pathway. NaB treatment reversed these abnormalities, whereas ANA‐12 completely abolished the therapeutic effects of NaB, indicating that activation of the BDNF/TrkB signaling pathway is a key mechanism underlying the neuroprotective effects of NaB.

Our in vivo findings indicate that the myelination abnormalities induced by sevoflurane exposure are not solely attributable to its direct toxic effects on OPCs, but may also be secondary to a reduction in the gut metabolite butyrate, which may serve as a key intermediary in sevoflurane‐induced developmental neurotoxicity. On this basis, we further dissected and validated this intermediary mechanism through in vitro experiments. Rather than directly modeling the direct effects of sevoflurane on OPCs at the cellular level, the present study employed exogenous NaB treatment of HOPCs to minimize interference from complex in vivo factors and thereby elucidate the direct regulatory effects of butyrate on OPCs. The results showed that the pro‐proliferative and pro‐differentiation effects of NaB on HOPCs were clearly concentration‐dependent. Within the low‐to‐moderate concentration range (100–500 μM), NaB significantly increased H3K9ac levels, promoted BDNF expression and TrkB phosphorylation, and further enhanced OPC proliferation and differentiation. However, at higher concentrations (≥ 1000 μM), NaB exhibited a certain biphasic effect, with attenuated protective efficacy, suggesting that excessively high concentrations may be accompanied by potential cytotoxicity. These findings indicate that there may be an optimal concentration range for NaB to exert its neuroprotective effects and provide an experimental basis for defining the therapeutic dosing window in future clinical translation. In addition, ChIP‐qPCR results further confirmed that NaB increased H3K9ac enrichment at the BDNF promoter region, promoted BDNF transcriptional activation, and thereby enhanced downstream TrkB signaling activity, ultimately supporting OPC function and myelin repair. These findings not only mechanistically support the hypothesis that butyrate deficiency is an important intermediary in sevoflurane‐induced neurodevelopmental injury, but also indicate that the in vitro model used in this study was not simply designed to replicate the in vivo exposure conditions, but rather to define the direct causal effects of key molecules. This experimental strategy helps isolate critical regulatory factors from complex in vivo effects, thereby providing clearer evidence for understanding the multilevel regulatory mechanisms underlying sevoflurane neurotoxicity and offering a new theoretical basis for the development of neuroprotective strategies targeting epigenetic regulation.

It is worth emphasizing that the present study is innovative in several respects. First, although the neuroprotective effects of butyrate have been previously reported, this study is the first to identify its key intermediary role in myelination impairment in the context of sevoflurane‐induced neurodevelopmental injury. Second, this study revealed the important role of the H3K9ac/BDNF/TrkB signaling axis in regulating OPCs' proliferation and differentiation, thereby integrating epigenetic regulation with neurotrophic factor signaling pathways and expanding our understanding of the biological mechanisms underlying OLs' function.

From a clinical translation perspective, the present findings suggest that NaB may have potential therapeutic value in alleviating anesthesia‐related neurodevelopmental injury. First, as a derivative of butyrate, NaB has good biocompatibility and low immunogenicity, and has been shown to exert neuroprotective effects in multiple animal models, suggesting a certain translational potential. Second, our study demonstrated that the effects of NaB on OPCs are clearly dose‐dependent and that NaB exhibits a biphasic effect at high concentrations, indicating that its effective dosage range and therapeutic window must be rigorously defined in clinical applications to avoid potential adverse effects. In addition, previous studies have shown that SCFAs can cross the blood–brain barrier (BBB) through monocarboxylate transporters (MCTs) [42], suggesting that NaB may have a certain degree of CNS accessibility. However, the permeability of the BBB and the effective intracerebral concentration of NaB at different developmental stages in neonates remain to be further investigated. Moreover, systematic studies on the combined use of NaB with existing anesthetic regimens are still lacking, and its safety in the perioperative setting, as well as its potential interactions with drugs used during anesthesia, require further in‐depth evaluation. Taken together, although the present study provides experimental evidence supporting NaB as a potential intervention strategy, its clinical translation still requires systematic pharmacokinetic studies, safety evaluations, and further preclinical and clinical validation.

Certainly, this study still has several limitations. First, this study was conducted using a rodent model. Although the model of repeated sevoflurane inhalation in neonatal mice has been widely used to simulate the effects of inhalational anesthetics on neurodevelopment [28], and mice share certain similarities with humans in terms of neural circuit formation and drug pharmacokinetics, inherent interspecies differences, particularly the substantial disparities in the developmental timeline of the nervous system and drug metabolic characteristics, may still limit the translational applicability of the findings. Second, clinical neonatal anesthesia typically involves the combined use of multiple drugs and diverse medical procedures. These complex factors may influence neurodevelopmental outcomes through synergistic or antagonistic effects, whereas current animal models cannot fully recapitulate such complicated clinical scenarios, thereby limiting the generalizability of the conclusions to some extent. In view of these limitations, we expect that future studies will develop more sophisticated animal models to more accurately replicate clinical conditions, thereby advancing a deeper understanding of the mechanisms underlying sevoflurane‐induced developmental neurotoxicity and facilitating the development of precise intervention strategies.

## 5 Conclusion

The present study demonstrated that repeated sevoflurane exposure during the neonatal period inhibits OPC proliferation and differentiation, impairs myelination, and ultimately leads to neurobehavioral deficits. Such exposure reduces butyrate production, resulting in altered H3K9ac modification, impaired chromatin accessibility at the BDNF promoter region, and disruption of normal BDNF/TrkB signaling. As a key regulatory pathway in neurodevelopment, BDNF/TrkB signaling plays a central role in maintaining myelination and neurobehavioral function.

## Author Contributions

**Chang Liu:** conceptualization, investigation, writing – original draft. **Xinyao Bai:** investigation, data curation. **Xinyue Liu:** methodology, formal analysis, writing – original draft. **Jinjie Li:** methodology, data curation. **Ruizhu Liu:** writing – review and editing, supervision, funding acquisition. **Jinlan Jiang:** writing – review and editing, resources. **Guoqing Zhao:** supervision, funding acquisition.

## Funding

This study was supported by the Innovation Capability Project of the Jilin Provincial Perioperative Organ Protection Engineering Research Center (Grant No. 3D518T743430) and the Jilin Provincial Natural Science Foundation (Grant No. YDZJ202601ZYTS528).

## Disclosure

*Transparency statement*: The design, data collection, analysis, and interpretation study adhere to rigorous scientific standards. All relevant data and methodologies have been clearly described in this manuscript, and any reproducible data are available upon request. There has been no selective reporting, and all hypotheses were based on a prespecified analysis plan.

## Ethics Statement

The animal protocol was reviewed and approved by the Animal Care and Use Committee of Jilin University (Changchun, China) (Ethical Protocol No.: SY: 2025‐03‐004). All animal experiments were conducted in accordance with the institutional guidelines for the care and use of laboratory animals.

## Conflicts of Interest

The authors declare no conflicts of interest.

## Supporting information


**Figure S1:** This figure shows the serum levels of five short‐chain fatty acids (SCFAs), including acetic acid, propionic acid, isobutyric acid, isovaleric acid, and valeric acid. It provides supplementary information on the trends of serum SCFAs shown in Figure 1D,E.
**Figure S2:** This figure shows the serum levels of five SCFAs, including acetic acid, propionic acid, isobutyric acid, isovaleric acid, and valeric acid. It provides supplementary information on the trends of serum SCFAs shown in Figure 3B,C.

**Table S1:** The antibodies used in this study.
**Table S2:** The antibodies used in this study.
**Table S3:** The specific primer pairs used in polymerase chain reaction.

## Data Availability

All data relevant to the study are included in the article and its Supporting Information, which are available from the corresponding authors upon reasonable request.
