# Supplementary material for: Sodium Butyrate Attenuates Sevoflurane‐Induced Impaired Myelination and Neurobehavioral Deficits in Neonatal Mice via the H3K9ac/BDNF/TrkB Pathway
Source: CNS Neurosci Ther. 2026 Jul 9;32(7):e71026. doi: 10.1002/cns.71026 (PMC13347314; doi:10.1002/cns.71026)
Supplement: Supplementary file 1 — Figure S1: This figure shows the serum levels of five short‐chain fatty acids (SCFAs), including acetic acid, propionic acid, isobutyric acid, isovaleric acid, and valeric acid. It provides supplementary information on the trends of serum SCFAs shown in Figure 1D,E. Figure S2: This figure shows the serum levels of five SCFAs, including acetic acid, propionic acid, isobutyric acid, isovaleric acid, and valeric acid. It provides supplementary information on the trends of serum SCFAs shown in Figure 3B,C. [file CNS-32-e71026-s002.docx]

**Figure S1**


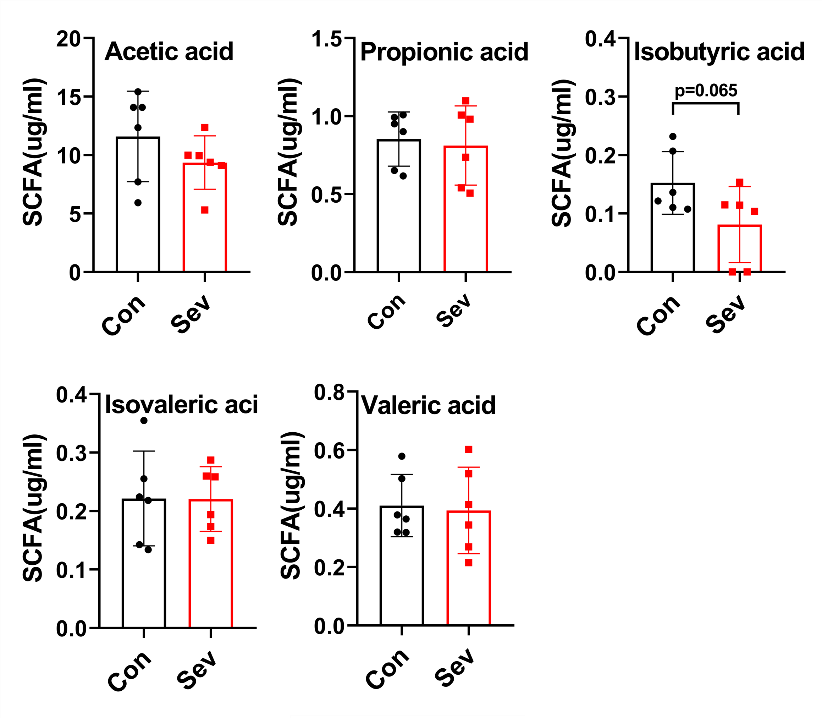


This figure shows the serum levels of five short-chain fatty acids (SCFAs), including acetic acid, propionic acid, isobutyric acid, isovaleric acid, and valeric acid. It provides supplementary information on the trends of serum SCFAs shown in Figures 1D-E.

**Figure S2**


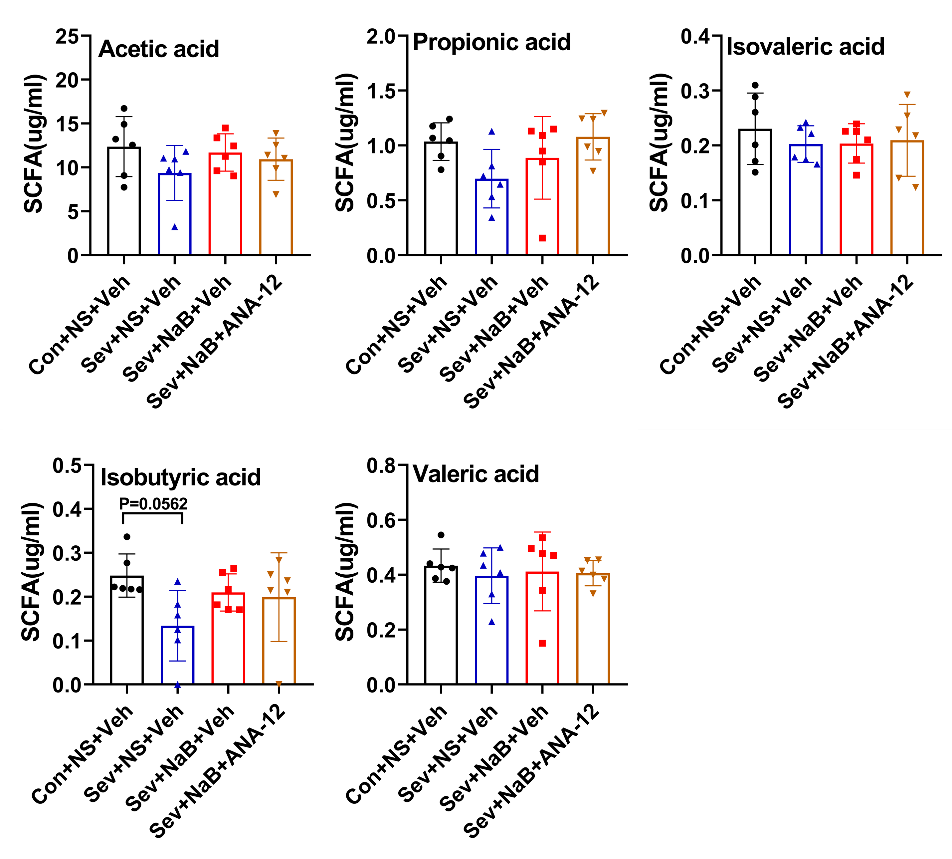


This figure shows the serum levels of five SCFAs, including acetic acid, propionic acid, isobutyric acid, isovaleric acid, and valeric acid. It provides supplementary information on the trends of serum SCFAs shown in Figures 3B-C.
