# Supplementary material for: Sodium Butyrate Attenuates Sevoflurane‐Induced Impaired Myelination and Neurobehavioral Deficits in Neonatal Mice via the H3K9ac/BDNF/TrkB Pathway
Source: CNS Neurosci Ther. 2026 Jul 9;32(7):e71026. doi: 10.1002/cns.71026 (PMC13347314; doi:10.1002/cns.71026)
Supplement: Supplementary file 2 — Table S1: The antibodies used in this study. Table S2: The antibodies used in this study. Table S3: The specific primer pairs used in polymerase chain reaction. [file CNS-32-e71026-s001.docx]

| **Table S1 The antibodies used in this study** | | | |
| --- | --- | --- | --- |
| Antibody | Dilution | Catalog No. | Source |
| PDGFRα | 1:500 | AB203491 | Abcam，UK |
| BrdU | 1:250 | AB6326 | Abcam，UK |
| Olig2 | 1:500 | OB-PRB009 | OasisBiofarm,China |
| CC1 | 1:200 | OB-PRB070 | OasisBiofarm,China |
| MBP | 1:100 | AB209328 | Abcam，UK |
| BDNF | 1:500 | AB108319 | Abcam，UK |
| P-TrkB | 1:100 | AF3461 | Affinity Biosciences,China |
| H3K9ac | 1:250 | AB32129 | Abcam，UK |
| Goat anti-rabbit IgG | 1:500 | AB150077 | Abcam，UK |
| Goat anti-rabbit IgG | 1:500 | AB150078 | Abcam，UK |
| Goat anti-mouse IgG | 1:500 | AB150113 | Abcam，UK |
| Goat anti-mouse IgG | 1:500 | AB150115 | Abcam，UK |
| Goat anti-Human IgG | 1:500 | AB7148 | Abcam，UK |

| **Table S2 The antibodies used in this study** | | | |
| --- | --- | --- | --- |
| Antibody | Dilution | Catalog No. | Source |
| PDGFRα | 1:1000 | AB203491 | Abcam，UK |
| Olig2 | 1:2000 | OB-PRB009 | OasisBiofarm, China |
| CNPase | 1:2000 | OB-PRT006 | OasisBiofarm, China |
| MBP | 1:1000 | AB209328 | Abcam，UK |
| P-TrkB | 1:1000 | AF3461 | Affinity Biosciences, China |
| TrkB | 1:1000 | AF6461 | Affinity Biosciences, China |
| BDNF | 1:1000 | AB108319 | Abcam，UK |
| HDAC1 | 1:1000 | AB280198 | Abcam，UK |
| HDAC2 | 1:2000 | AB32117 | Abcam，UK |
| HDAC3 | 1:5000 | AB32369 | Abcam，UK |
| HDAC8 | 1:1000 | AB274372 | Abcam，UK |
| H3K9ac | 1:500 | AB32129 | Abcam，UK |
| Total H3 | 1:500 | AB150113 | Abcam，UK |
| β-actin | 1:5000 | AF7018 | Affinity Biosciences, China |
| α-Tubulin | 1:1000 | AF4651 | Affinity Biosciences, China |

| **Table S3 The specific primer pairs used in polymerase chain reaction.** | | |
| --- | --- | --- |
| Gene | Forward (5′-3′) | Reverse (5′-3′) |
| Sox10 | ACGGTTTTCCACTTCCTCA | GTCTTGTTCCTCGGCCAT |
| Myrf | TCAGAGGCCATCGTTCAG | CCCAGTCAAGGGAAGCA |
| BDNF | GCTCGAAGTCAGATCCACA | GCATCCTCAGCCCCTAC |
| BDNF Primer1 | GCTGGAGGTAACTGTTGCCTTGG | GCTCTTGGTGACCTGGTGTGC |
| BDNF Primer2 | CCACCATCTTCCGCAACATCTCC | GACCCCACCAAAGGCATCACTG |
| BDNF Primer3 | ACACGCGCACACACATACGG | CAGGCTGGGAAGTGGAGAGGTC |
| Actb | TCTTTGCAGCTCCTTCGT | GACCCATTCCCACCATC |
